# Nudging enforcers: how norm perceptions and motives for lying shape sanctions

**DOI:** 10.1093/pnasnexus/pgad224

**Published:** 2023-07-04

**Authors:** Eugen Dimant, Tobias Gesche

**Affiliations:** Center for Social Norms and Behavioral Dynamics, University of Pennsylvania, Philadelphia 19104, USA; CESifo, Munich 81679, Germany; Center for Law & Economics, ETH Zurich, Zürich 8092, Switzerland

**Keywords:** norm-nudges, nudging, social norms

## Abstract

We conduct three studies, employing diverse methodologies (a behavioral experiment, a vignette experiment, and a norm elicitation experiment), to investigate when and how norm enforcement patterns can be modified using norm interventions in the context of dishonesty. Our preregistered, three-part data collection effort explores the extent to which norm violations are sanctioned, the impact of norm-nudges on punishment behavior, and the connection to norm perception. Using a representative sample of US participants in Study 1, we present robust evidence that norm enforcement is sensitive not only to the magnitude of the observed transgression (i.e. the size of the lie) but also to its consequences (whether the lie addresses or creates payoff inequalities). We also find that norm enforcers respond to norm-nudges conveying social information about actual lying behavior or its social disapproval. The results of a separate vignette experiment in Study 2 are consistent with the results in our behavioral experiment, thus hinting at the generalizability of our findings. To understand the interplay of norms, information about them, and punishment, we examine norm perceptions across different transgressions in Study 3. We find that norm perceptions are malleable and norm-nudges are most effective when preexisting norms are ambiguous. In sum, we show how norm enforcement can be nudged and which factors matter for doing so across various contexts and discuss their policy implications.

**Significance Statement**

This research examines how to positively influence behavior by subtly guiding people’s understanding of what’s considered socially acceptable—a method referred to as ”norm-nudging”. In several studies, we find that people’s norm enforcement behavior is sensitive to such norm-nudges: we find that their willingness to punish dishonesty does not just depend on how big the lie is, but also on whether the lie creates or remedies unfairness. With that, we show that these norm-nudges can amplify the tendency to punish norm violations. We also discovered that these nudges work best when existing norms are unclear. Our takeaway is that norm-nudging can complement top-down regulatory approaches, and offer a more nuanced strategy for sustaining norm adherence.

## Introduction

Social norms are ubiquitous in human societies. They inform individual behavior in social and economic domains such as collective action, altruistic sharing, and deviance.^a^ The existing consensus is that norm compliance can erode quickly, and enforcement is crucial to sustaining social order (1–7).

A particularly promising approach to enact behavioral change has emerged in the form of nudging (8, 9). The existing nudge literature typically focuses on interventions that aim to change the behavior of transgressors directly (e.g. Refs. (10–16)). Recently, a growing body of literature has utilized the so-called “norm-nudges”: interventions that attempt to change behavior by eliciting and changing existing social norms through the manipulation of social expectations. As conceptualized by Bicchieri and Dimant (17, 18), interventions that aim to change beliefs about what others in one’s reference network *do* (descriptive element of the norm, first-order belief) or *approve* of others doing (injunctive element of the norm, second-order belief).^b^

The existing literature has primarily focused on the drivers of punishment in strategic and cooperative settings (e.g. Refs. (19, 20)), with limited exploration of how punishers’ norm enforcement behavior can be influenced by norm-nudges. Our study specifically investigates the impact of norm-nudges on norm enforcement in *non*strategic contexts (i.e. a lack of an interactive component in the punishment process, specifically the absence of potential retaliatory measures by the potential liars). These settings feature transgressions that systematically differ in magnitude and consequence, enabling us to isolate confounding factors related to ulterior motives, such as enforcing punishment to increase one’s own payoff. We achieve this by measuring norm enforcement through the lens of third-party punishment that is not instrumental within the one-shot experimental environment (21). Although previous research highlights instances where norm violators and those who fail to punish them are punished (22, 23), there is—to the best of our knowledge—no systematic examination of how breaching different types of norms influences enforcement. Our core contribution lies in demonstrating when and how norm enforcement patterns can be altered using norm-nudges. We find that nudging in environments with less clearly defined norms results in more significant shifts in norm perceptions than in settings with well-established a priori norms (24, 25).

Our study also illuminates potential mechanisms that can explain observed real-world normative and behavioral shifts. Take, for instance, the campaigns against indoor smoking. In the early phases, the focus was on individual health risks associated with smoking. Over time, the strategy evolved towards emphasizing the social unacceptability of smoking indoors, effectively leveraging the “norm-nudge” mechanism (26). This evolution in strategy marked a transition from attempting to change individual behaviors to influencing societal norms about indoor smoking, with the intention of harnessing social pressure and peer sanctioning to enforce compliance. The insights we present in our studies echo this shift, suggesting that a change in focus from addressing individual transgressors to modifying the broader societal norms can lead to significant alterations in behavior.

We contribute to the literature by offering an approach that complements traditional nudging methods. Instead of focusing solely on directly nudging transgressors, we harness the power of norm-nudges to examine their effects on those who *enforce* norm compliance through punishment. We evaluate the effectiveness of interventions using social information to guide norm enforcement across various decision scenarios with differing motives and degrees of norm breach. This allows us to assess interventions designed to nudge individuals in positions of power, who can enforce adherence to social norms among transgressors. To achieve this, we carried out three well-powered, preregistered studies that were run with three separate sets of participants (no participant took part in more than one study): *Study 1* represents our main, incentive-compatible behavioral experiment. *Study 2* is a vignette experiment that offered a flat participation fee to assess the robustness of Study 1’s behavioral findings in a different context. *Study 3* is an incentive-compatible norm elicitation experiment that investigates the relationship between norm perceptions and punishment patterns observed in Study 1.

Study 1 examines how individuals adjust punishment behavior based on different motives for noncompliance with a norm. We investigate how punishment varies when the consequences of a lie differ, such as lying to gain an unfair advantage versus lying to restore equal chances. In our setting, a norm enforcer (*punisher*) is a third-party that observes the lying behavior of another participant where that person (*liar*) can misreport the outcome of a die roll to gain an unfair advantage over another person (*victim*). We then explore the influence of norm-nudging by examining the sensitivity of punishment decisions of the third-party norm enforcer to empirical information (what others *do*) and normative information about others’ actions (what others *approve of doing*). We borrow this approach directly from the social norms literature (e.g. Refs. (27, 28)). We find that participants’ punishment decisions are affected not only by the size and outcome of the lie but also by the norm-nudge they receive. We confirm the robustness of our findings in Study 2 using a vignette experiment focused on corruption in a company setting. The results are consistent with those from Study 1. In Study 3, we find that elicited norm perceptions align with Study 1 results: lying that benefits the liar relative to another participant is perceived as less appropriate than lying that creates more equal chances for both participants. Moreover, we observe that providing norm information (i.e. normative statements about the inappropriateness of lying or information that a majority of others did not lie when given the opportunity) enhances the perceived inappropriateness of lying, regardless of the information source. We find evidence that providing norm information is particularly effective in situations where existing norms can justify another norm breach (i.e. lying is perceived as less punishable when it results in more equal chances for both participants).

Taken together, our experiments allow us to investigate *when* and *how* norm breaches are sanctioned; the results also suggest both that variations in norm enforcement behavior are consistent with variations in norm perceptions and that these norm perceptions are malleable depending on the motives for lying. Intuitively, this suggests that regulatory (top-down) interventions implemented to change behavior can be complemented by social (bottom-up) enforcement through informal norm-nudging, at least where social norms are clearly defined, transgressions are observable, and when they can be sanctioned. With that, we connect three literature streams: the study of transgressions in the context of lying, the enforcement of norms through punishment, and the perception of social norms. We contribute to the existing literature on nudging by investigating ways to improve norm enforcement with social rather than monetary stimuli for enforcers, which is an alternative to traditional policy interventions that focus on nudging the behavior of transgressors directly (29).

## Study 1: Norm information and norm enforcement

### Experimental design and procedures of the behavioral experiment

**Fig. 1.**
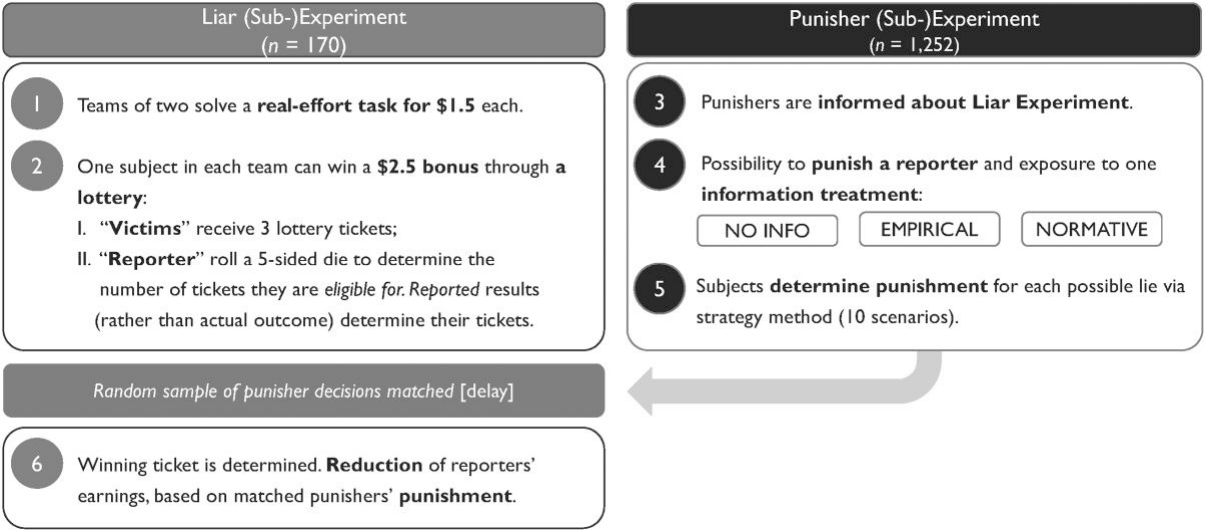
Design of the behavioral experiment in Study 1.

Study 1 consists of two subexperiments: a *Liar Experiment* and a *Punisher Experiment*. The former is necessary to elicit punishment behavior in the latter in an incentive-compatible manner. Fig. 1 provides an overview of the main steps of these experiments, which were carried out in April/May 2019. A detailed description follows:

#### Step 1—Real-effort task

The Liar Experiment involved n=170 participants, recruited via Amazon’s Mechanical Turk (MTurk); their demographics are presented in Table S1 in the online supplementary material.^c^ Participants were grouped in teams of two. Each team member then had to solve a real-effort task by correctly counting the 1s in five matrices with numbers. This took about 7 min and earned each team member $1.5 (added to a base payment of $0.5).

#### Step 2—Bonus lottery and lying

Subjects in the Liar Experiment knew that in addition to their individual compensation of $1.5, solving the real-effort task entitled them to win an added bonus of $2.5. However, this bonus could only be won by one person within each team. Specifically, the bonus was allocated by drawing a winning lottery ticket for each team where the number of tickets (and therefore chance of winning) differed by a subject’s role:

- *Victim*: One of the two subjects in each team was randomly allocated to this role and received a fixed amount of three lottery tickets.
- *Reporter*: The other subject on the team was entitled to a number of lottery tickets equal to the randomized outcome of a virtual 5-sided die that they rolled. However, subjects in this role were made aware that what they *reported* (rather than the actual outcome) would determine the number of lottery tickets they would receive.

Our design created an incentive for reporters to exaggerate the outcome of their die roll, thereby enhancing their odds of winning the bonus. This incentive aligns with the “die under the cup” paradigm (analogous to Fischbacher and Föllmi-Heusi (30), with the notable distinction that we had observable outcomes). Importantly, this setting is nonstrategic by design. Reporters who lie cannot rationalize their actions by forming (motivated) beliefs about their teammate’s responses since they do not have the option to lie. In order to accurately measure dishonest behavior while avoiding deception, reporters were informed post-submission that a third participant, the *punisher*, would later observe—and potentially penalize—their behavior, with penalties up to $1.50. Reporters were then offered the chance to modify their initial reports, understanding that only upward lying could be subject to punishment. The revised report served as the basis for their punishment in the behavioral experiment. This protocol remained consistent across all treatments in the punisher experiment, as outlined below.

#### Step 3—Punishers learn about the Liar Experiment

The Punisher Experiment featured n=1,240 subjects. They were recruited through a professional market research firm in order to get a representative sample of the US working-age population (for subjects’ demographics, see Table S2 in the online supplementary material). This allows us to draw more generalizable inferences (see Levitt and List (31)).^d^ In the first part of the Punisher Experiment, the subjects read a description of the lying game. They were then assigned to the role of a punisher and learned that they would be matched with a reporter from the Liar Experiment whom they could punish for dishonest behavior. Punishment was only based on the revised report by liars (see Step 2) and liars knew about that. Punishers also had to pass comprehension questions first to ensure that they understood the context and impact of their decision.

#### Step 4—Norm information treatments

Right before punishers were asked to determine their punishment, they were presented with one randomly assigned norm information treatment. The two treatments (EMPIRICAL and NORMATIVE) provided norm information, whereas the baseline condition (NO INFO) contained no such information. The provided information was either descriptive (what other participants previously *did*; treatment EMPIRICAL) or normative (what other participants stated is the right thing to do; treatment NORMATIVE). More precisely, punishers in EMPIRICAL were told about a previous, auxiliary study in which a “Player A” was in the same decision scenario as reporters in the Liar experiment and what Player As *actually did*. Punishers in NORMATIVE were told about another auxiliary study in which participants said what Player As *should* do.^e^ One of the following two messages was then presented to punishers right before punishment decisions could be made:

Norm-nudge in EMPIRICAL—what Player As (decision in Liar Experiment) *actually did:*

***The majority of Player As in the previous study reported the number truthfully (i.e., reported exactly what the die showed).***

Norm-nudge in NORMATIVE—what others stated Player As *should do:*

***The majority of these people stated that the right thing to do for Player A is to report the number truthfully (i.e., report exactly what the die showed).***

#### Step 5—Punishment

Punishment was elicited via a strategy method: For each of the 10 possible scenarios of dishonest reporting in the Liar Experiment depicted in Table 1, punishers could assign between 0 and 5 punishment points. Punishers knew that if they were matched with a liar, their decision would have direct payoff consequences for that liar. That is, the liar’s earnings would be reduced by $0.3 for each punishment point that the punisher assigned in the particular scenario that corresponded to the actual behavior of the liar.^f^ Our experimental design intentionally prevents counter-punishment to ensure our results focus on the channels of interest. By doing so, we can eliminate the influence of subjects’ higher order beliefs about others’ actions and concentrate on studying the effects of transgression motives and the provision of norm-nudges.^g^ Table 1 shows an example of how a punisher could assign punishment.

**Table 1.** Assignment of punishment points (example).

*I want to assign the following number of punishment points if Participant A…*						
	*…had a die outcome of “**1**”*					
	0	1	2	3	4	5
*…and reported “**2**”*	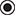	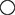	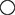	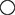	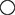	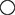
*…and reported “**3**”*	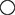	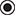	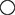	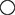	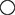	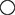
*…and reported “**4**”*	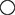	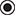	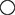	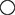	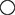	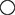
*…and reported “**5**”*	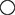	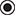	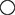	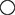	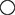	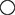
	*…had a die outcome of “**2**”*					
	0	1	2	3	4	5
*…and reported “**3**”*	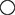	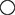	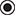	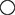	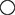	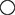
*…and reported “**4**”*	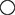	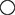	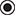	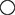	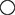	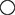
*…and reported “**5**”*	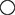	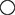	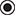	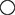	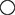	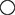
	*…had a die outcome of “**3**”*					
	0	1	2	3	4	5
*…and reported “**4**”*	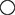	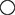	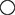	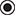	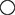	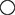
*…and reported “**5**”*	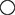	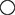	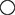	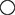	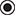	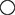
	*…had a die outcome of “**4**”*					
	0	1	2	3	4	5
*…and reported “**5**”*	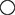	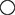	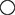	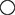	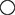	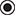

*Note*: The order determining whether punishment scenarios were presented by the die’s actual outcome in an increasing manner (shown here) or decreasing manner was randomized. For punishers, a reporter was referred to as “Participant A.” For the original screen, see online supplementary material D. The lying/punishment scenarios presented here will be referred to as p12, p13, p14, p15, p23, p24, p25, p34, p35, and p45, respectively.

#### Step 6—Matching and payment

The experiment ended after the punishers made their decisions and answered an exit questionnaire. Subsequently, a subset of punishers was randomly chosen to be matched with the reporters from the lying experiment. We also elicited punishers’ beliefs about the chances of matching with a liar. We control for these beliefs in all relevant regressions in the results section.^h^ Based on their matched counterpart choices, participants were then paid their respective earnings and bonuses.^i^

#### Further design aspects

Our norm information intervention (EMPIRICAL or NORMATIVE, see Step 4) builds upon a well-established tradition in the social norms literature. This body of work has consistently validated the use of “majority” messages, which employ the majority of others’ behavior or approval to highlight social norms (see, e.g. Refs. (11, 32, 33)). Social norms are understood as behavioral patterns rooted in a shared understanding of acceptable actions within a reference group (34). They comprise two distinct components (see Refs. (27, 28)): an empirical component (often referred to as *descriptive norm*) and a normative component (often referred to as an *injunctive norm*). Norms can thus be seen as coordination games among reference group members, with shared signals sustaining norm adherence by facilitating coordination (27, 35). Previous research has demonstrated that both social norm components influence and direct behavior, though their relative effectiveness often varies (3, 11, 36–39). This distinction is integral to our experimental design, allowing us to capture the heterogeneous effects of norm-nudges depending on the “component” of a norm that is violated.

To accurately assess the efficacy of norm-nudges, we employed the strategy method, eliciting punishment behavior for each potential lying scenario. As Brandts and Charness (40) argue, each method has its advantages and drawbacks. Their evaluation of punishment studies reveals that the strategy method generally yields lower effects compared to the direct response approach, where punishers respond to concurrent lying behavior. Consequently, our findings likely represent a lower-bound effect of norm-nudges. Note that our consistent application of the strategy method across all scenarios for all subjects ensures any inherent demand effect inherent does not correlate with our norm-nudge treatments (see supporting discussion in Refs. (41, 42)). Another crucial aspect of our design is that punishing is costless for the punisher, both directly and indirectly (i.e. no risk of counter-punishment). This design choice allows us to exclude confounding factors such as an additional monetary trade-off, image concerns, or risk assessment (43, 44). Our focus is to evaluate the potency of norm-nudging in shaping punishment patterns, void of any incentives that could potentially counteract the nudge intervention. While we acknowledge that punishment costs influence behavior (see, e.g. Refs. (45, 46)), incorporating such punishment would have introduced another layer of complexity. Moreover, its *direct* effect would have been uniform across all treatments, thus not influencing any observed treatment differences. This renders it an inconsequential design choice given our research objectives. Lastly, we recognize that norm-nudges, appealing to a social norm, might be *inherently* perceived by subjects as an experimenter demand. From an observational standpoint, this particular demand effect could be interpreted as subjects adhering to norms. However, studies that aim to induce such artificial experimenter demand effects only succeed when using strong language (e.g. requesting to “do us a favor,” see De Quidt et al. (41)). Our study design deliberately avoids this, adhering to best practices outlined in De Quidt et al. (47).

### Behavioral predictions

In Study 1, the design of the die roll and reporting task in the Liar Experiment facilitated the creation of diverse “punishment scenarios” for the Punisher Experiment. We designate these scenarios using the prefix “p” followed by a two-digit number. The first digit represents the actual outcome of the die roll, while the second digit signifies the reported outcome. Hence, a scenario wherein the active player rolled a “1” but reported a “2” is denoted as “p12.” These 10 scenarios vary principally along two dimensions:

1. The size of the lie (how much the reported outcome of the die roll exceeded the actual one).
  - *Lie size = 1:* report larger by 1 than actual outcome (p12, p23, p34, and p45).
  - *Lie size = 2:* report larger by 2 than actual outcome (p13, p24, and p35).
  - *Lie size = 3:* report larger by 3 than actual outcome (p14 and p25).
  - *Lie size = 4:* report larger by 4 than actual outcome (p15).
2. The “equity nature” of a scenario (the chances of obtaining the bonus for the *active* player, relative to the *passive* player).
  - *Equity:* lying leads to more equity (reduces the gap) in the chances of winning the lottery (p12, p13, and p23).
  - *Inequity:* lying leads to (more) inequity in the chances of winning the lottery (p34, p35, and p45).
  - *Overclaiming:* starting from a situation with a disadvantaged active player, lying *reverts* inequality, leading to a now disadvantaged *passive* player (p14, p15, p24, and p25).

Combined with the norm information treatments, these punishment scenarios allow us to extend insights from the existing literature to explore the relationship between motives for norm breach and punishment. This led to the following set of preregistered hypotheses (see online supplementary material C).

#### Size of the lie

We start by drawing on the extensive literature in the social sciences, which has established the determinants of lies and lying costs (48–51). Little, however, is known about how these findings are reflected in the punishment of lies, especially within the context of social norm breaching. Based on existing theories and experimental evidence in regard to lying, we hypothesize that not only the occurrence of a lie but also its size matters:

Hypothesis 1: *The amount of punishment assigned increases with the size of the lie, which can be perceived as the reported outcome minus the actual outcome.*

#### Equity nature of the lie

A large amount of literature emphasizes the importance of equity concerns, including in the context of deviant behavior (52–54). However, an unexplored question is whether such motivations matter for the assessment of a norm breach and, consequently, affect the severity of punishment. However, a mere aversion to unequal chances of getting the bonus is not enough to explain the hypothesized effects of a norm-nudge. This is because the norm-nudge is against lying *in general* and across our 10 punishment scenarios, lying, on average, does increase inequity in such situations just as much as it decreases it. This is evenly balanced in the size of the lie, which is why such an aversion does not explain the effect of the lie’s size on punishment.^j^ However, for a given lie size, some punishment scenarios do increase this inequity while others decrease it. We hypothesize that breaching a norm in the form of overreporting for the purpose of “getting ahead unfairly” is assessed differently from such breaching for the purpose of leveling the playing field (see also Refs. (55, 56)). While this logic also leads us to expect that lying in Inequity-scenarios or Overclaim-scenarios will be punished harsher than in the Equity-scenarios, how punishments differ between the former two scenarios is an empirical question that we will investigate in our analysis.

Hypothesis 2: *The equity nature of the lie matters. For a given size of the lie, the amount of punishment assigned*

- *in Equity-scenarios is lower than in Inequity-scenarios,*
- *in Equity-scenarios is lower than in Overclaim-scenarios.*

#### Effect of norm-nudges

Finally, we derive hypotheses for our norm-information treatments. Existing research suggests that people are receptive to norm information and conform to both observed behavior and normative messaging (e.g. Refs. (57–59)). Therefore, we hypothesize that norm-nudges in the EMPIRICAL and NORMATIVE treatments lead to more punishment compared to those in NO INFO. Furthermore, theoretical and experimental insights from Bicchieri et al. (37) suggest that while people interpret honest behavior as a strong indicator of normative disapproval of lying, the reverse may not be true: Merely saying what (not) to do does not necessarily have to be followed by the corresponding actions. Thus, we expect that empirical information may work as a stronger norm-nudge, where acting in breach of what people actually do (“walking the talk”) can be a stronger signal than acting in breach of what people say one should do:

Hypothesis 3: *The amount of punishment assigned increases over our three treatments in the following order: NO INFO < NORMATIVE < EMPIRICAL.*

### Results of the punishment experiment

Here, we report the actions of the punishers in Study 1. In doing so, we first look at lies and then at their punishment, following the order of the hypotheses described above using nonparametric methods. In online supplementary material A, we corroborate our results using a regression framework.

#### Lies

Given the incentive to lie when reporting the die outcomes, we find that reported outcomes were indeed on average about 29% higher than the actual outcome of the die roll (Wilcoxon signed rank test: P<0.001). Punishment was also calibrated well enough in that the *threat of* punishment works—revised reports were, on average, lower by 0.44 than the initial reports (signed rank test: P<0.001). However, even revised reports were still higher (by about 0.46) than the actual outcomes (signed rank test: P=0.003). We also observe relatively little downward lying: 2.4/3.7% of the subjects lied downwards in the first/revised report. Fig. S1 in the online supplementary material provides more details on subjects’ reporting patterns.

#### Punishment and the size of the lie

**Fig. 2.**
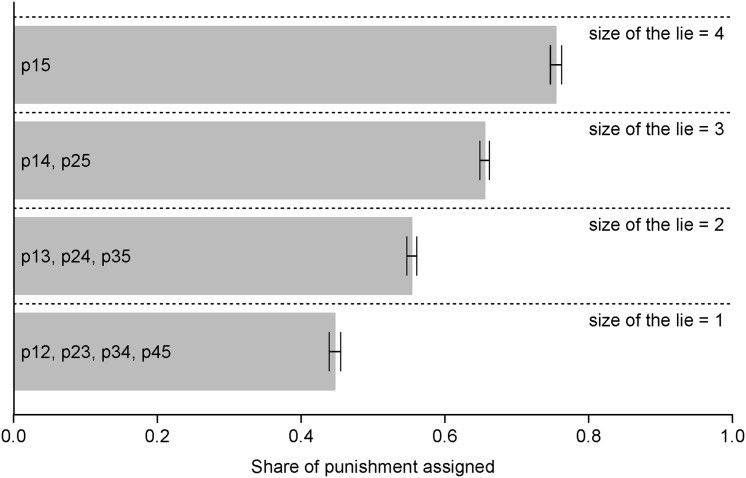
Punishment by size of the lie. *Note:* Punishment is assigned as a share of total punishment points available in the punishment scenarios for a given size of the lie (associated punishment scenarios are displayed in each bar). Error bars denote standard errors of the mean.

To examine the relationship between the size of the lie and punishment, we calculate the share of punishment assigned across punishment scenarios across different sizes of lies. In doing so, we can compare punishment even if the number of underlying punishment scenarios and, thus total possible punishment, differs across lie sizes. Fig. 2 visualizes the results (Fig. S2 in the online supplementary material shows the ungrouped results for every single punishment scenario). Consistent with Hypothesis 1, the punishment increases significantly with the size of the lie.

Specifically, we observe that the smallest possible lie (size = 1) is only assigned a share of punishment of 44.7% of the maximum possible punishment. With each larger lie, the share of the punishment increases by about 10 percentage points linearly. A Wilcoxon signed rank test also shows that the punishment shares for the different sizes of lies always differ significantly (P<0.001 for all six pairwise comparisons). These results show that individuals do not only punish norm breaches per se but also consider the extent to which norms are breached.

#### Punishment and the equity nature of the lie

Hypothesis 2 posits that while individuals care about the extent of a norm breach, not all norm breaches are created equally and, thus, punished equally. That is, lying to correct an initial unfair situation might be judged—and punished—differently than lying to exacerbate an already unfair situation. Consequently, we examine Equity-, Inequity-, and Overclaim-Lying separately.

Fig. S4 in online supplementary material A displays the average punishment level for each individual punishment scenario. In both cases, there are three associated scenarios with the same lie sizes (2 × Lie size = 1 and 1 × Lie size = 2). While the share of punishment for lies that achieve equity is 43.6%, this share for lies that achieve inequity is 9.2 percentage points higher, at 52.8%. We also find that the punishment choices across these two equity norms are significantly different (Wilcoxon signed rank test: P<0.001). This confirms Hypothesis 2a.

**Fig. 3.**
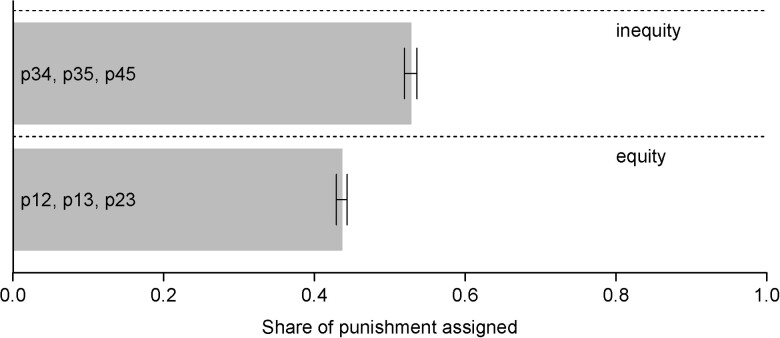
Punishment by equity norm. *Note:* Punishment is assigned as a share of total punishment points available in the punishment scenarios for a given equity norm (associated punishment scenarios are displayed in each bar). Error bars denote SEM.

Note that the figure does not display punishment for Overclaim-Lying (p14, p15, p24, and p15) as these scenarios are more numerous and feature higher sizes of the lie than the above one. To account for that, we employ a regression framework. This allows us to explicitly control for the fact that the four Overclaiming-scenarios (p14, p15, p24, and p25) entailed larger lies than the three Equity- and the three Inequity-scenarios presented in Fig. 3. The regression results are presented in Table S4 in online supplementary material A. Those results show that even with these controls, punishment is higher in the Overclaiming-scenarios than in the Equity-scenarios (5.1%; *t*-test P<0.001), thus confirming Hypothesis 2b.

#### Punishment and norm-nudges

**Fig. 4.**
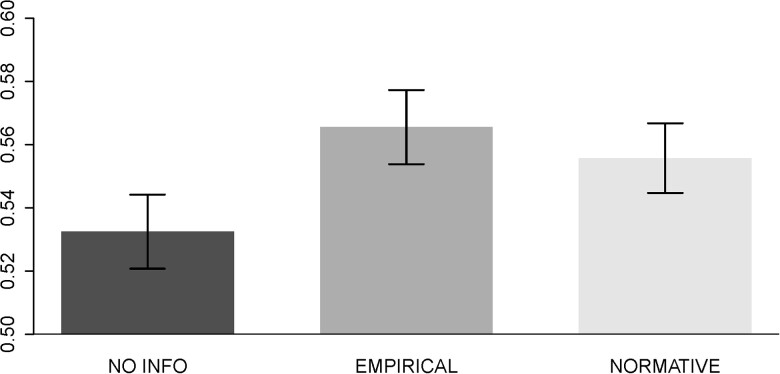
Punishment in the different norm information treatments. *Note:* Punishment is assigned as a share of total punishment points available in the 10 punishment scenarios, by norm-information treatments. Error bars denote SEM.

To examine Hypothesis 3, we start by looking at the aggregate impact of norm-nudges on punishment. We do so by computing the share of the total punishment which punishers could assign over the 10 identical punishment scenarios they all faced. We then compare that share over the three norm-info treatments punishers were distributed across. Fig. 4 displays the corresponding means and their associated standard errors.

Following Hypothesis 3, we expect behavior that is in conflict with others’ behavior (EMPIRICAL treatment) to lead to a punishment that is at least as harsh as behavior that is in conflict with what is deemed appropriate by others (NORMATIVE treatment). In fact, we observe the highest punishment in the EMPIRICAL treatment, which is significantly different from punishment in the NO INFO treatment (Wilcoxon rank-sum test: P=0.038). Punishment in EMPIRICAL is also directionally—but not significantly—larger than punishment in the NORMATIVE condition (Wilcoxon rank-sum test: P=0.734). Our results also show that punishment in the NORMATIVE condition is larger than punishment in the NO INFO condition (Wilcoxon rank-sum test: P=0.058). In summary, we find evidence that is consistent with parts of Hypothesis 3: receiving norm- info leads to higher punishment of lying behavior. However, we do not find a significant difference in that the empirical nature of such information has a stronger impact than mere normative assertions.

The regression in online supplementary Table S4 also allows us to re-test our other hypothesis with additional controls for punisher characteristics and beliefs. None of its results changes the insights from the nonparametric test above, therefore re-confirming Hypotheses 1, 2, and parts of 3.

## Study 2: Corroborating Study 1 using a vignette experiment

In order to assess the robustness and external validity of our main findings from Study 1, we conducted a vignette experiment featuring multiple treatments and random assignment. We designed the vignette to resemble a real-life situation while maintaining key elements from the original behavioral experiment: bribe acceptance (akin to cheating) and whistle-blowing (similar to punishing) in a corporate context. Our working assumption posits that testing our main insights—punishers are sensitive to both the deviant behavior’s motive and the presence of norm information—in a distinct context and methodology is an initial step toward enhancing external validity (for a related discussion, see List (60)). We collected a general population sample of n=225 in person through research assistants across 10 US states.^k^ Subjects were randomly presented with a scenario in which they observed a coworker accepting a bribe and were given the opportunity to blow the whistle by submitting incriminating evidence about the bribe-taking behavior. They could choose among different actions (submitting varying amounts of evidence) and thus influence the likelihood of their coworker facing punishment.

The vignette experiment employed a 2(within) × 2(between)-design, mirroring Study 1’s treatment dimensions (see online supplementary Fig. S6). The within-subject conditions, presented in a random order, focused on the (in)equitable nature of the coworker’s bribe acceptance: either creating EQUITY relative to the subject’s role (the coworker had not received an end-of-year bonus due to external factors, unlike the subject) or creating INEQUITY (the coworker received the same bonus as the subject, with the bribe in addition). Furthermore, the between-subject variation determined whether subjects received norm-nudge information, as in the behavioral experiment’s NORMATIVE condition (information about the majority of participants in a previous study objecting to bribe acceptance), or no information as in the NO INFO condition. online supplementary material B contains further details on the vignette experiment’s design and results, as summarized below.

We find that providing norm information increases the average chosen probability of punishment over equity-scenarios overall (75.0% vs. 65.7% in INFO and NO INFO, respectively; P≤0.044 in regression and rank-sum tests; see also online supplementary Fig. S7). This effect of providing norm information appears in both, the INEQUITY-scenario (82.8% vs. 73.3%; P<0.005) and the EQUITY-scenario (67.2% vs. 57.9%; P=0.074). Likewise, we also repeat our observation from the behavioral experiment in that, overall, the chosen average punishment for opportunistic behavior is higher if it creates INEQUITY rather than EQUITY (77.8% vs. 62.3%; P<0.001). Together, we confirm the findings of the behavioral experiment and show that the punishment patterns observed in Experiment 1 replicate in an applied situations, such as whistle-blowing in the workplace.

## Study 3: Norm information and norm perception

The findings from Study 1 indicate that people’s punishment of norm violations varies according to the norm information they receive and the influence of the lie on (in)equity. To better understand why norm enforcement differs in these cases, we explore whether the observed punishment patterns correspond to variations in the perception of social norms. This investigation is crucial because long-lasting adherence to norms relies on enforcement reflecting shared (perceived) social norms, and will likely be less effective if the norms conflict with formal rules or represent idiosyncratic judgments (3, 61).

Previous research demonstrates that providing norm information can, but does not necessarily, alter social norm perceptions (see, e.g. Refs. (62, 63)). Therefore, determining whether changes in punishment patterns align with shifts in social norm perceptions is an empirical question within our experimental context. We designed Study 3 to examine this by varying the type of norm information and the equity nature of lies, as in Study 1. Using a new set of participants, we employ the standard norm elicitation procedure by Krupka and Weber (64) to assess how social norm perceptions differ across these dimensions, rather than focusing on punishment decisions. This procedure has been used to study norms across various settings, including lying contexts similar to ours (63).^l^ For norm elicitation to be informative, incentivization in this task does not need to match, and usually does not, the incentivization in the task where social norms are elicited. The key concept of this method is to elicit incentive-compatible norm beliefs across the original settings of the behavioral experiment, which are thoroughly explained to participants. They then evaluate the social (in)appropriateness of lying behavior in those original settings.

### Design

In November 2019, we recruited a new set of n=1,519 subjects through MTurk to obtain their norm perceptions of lying behavior as observed in the behavioral experiment of Study 1. We elicited perceptions regarding the normative appropriateness of lying for those scenarios using the incentive-compatible procedure by Krupka and Weber (64).

Before eliciting their norm perceptions, participants were informed of the original lying task in the same way that it was explained to punishers in the behavioral experiment of Study 1 (i.e. about the structure of Part 1 and Part 2; see Fig. 1). Subsequently, each subject was presented with one of several lying situations that varied along two main dimensions. The first dimension was whether no norm information (NO INFO), normative information (NORMATIVE) or empirical information (EMPIRICAL) was provided on top of the observed lying behavior. The second dimension was the equity nature of the lie, which corresponds to three lying scenarios reflecting Equity-scenarios (p13), Inequity-scenarios (p35), and Overclaim-scenarios (p24). Note that for comparability, the size of the lie was constant (at a size of 2). This yields a between-subjects design that varies norm information and equity nature of a lie in a fully factorial manner over 3 (norm information) × 3 (equity lying scenarios) treatments to which subjects were randomly assigned.^m^ We measured our dependent variable of interest by asking participants to rate the extent to which other subjects deemed the observed lying behavior socially (in)appropriate. They did so using a 4-point Likert scale ranging from “very socially appropriate” (*VSA*) over“somewhat socially appropriate” (*SSA*) and “somewhat socially inappropriate” (*SSI*) to “very socially inappropriate” (*VSI*). In each treatment, participants were given a monetary incentive to guess the modal answer, allowing incentive-compatible elicitation of norm perceptions.

### Results

**Fig. 5.**
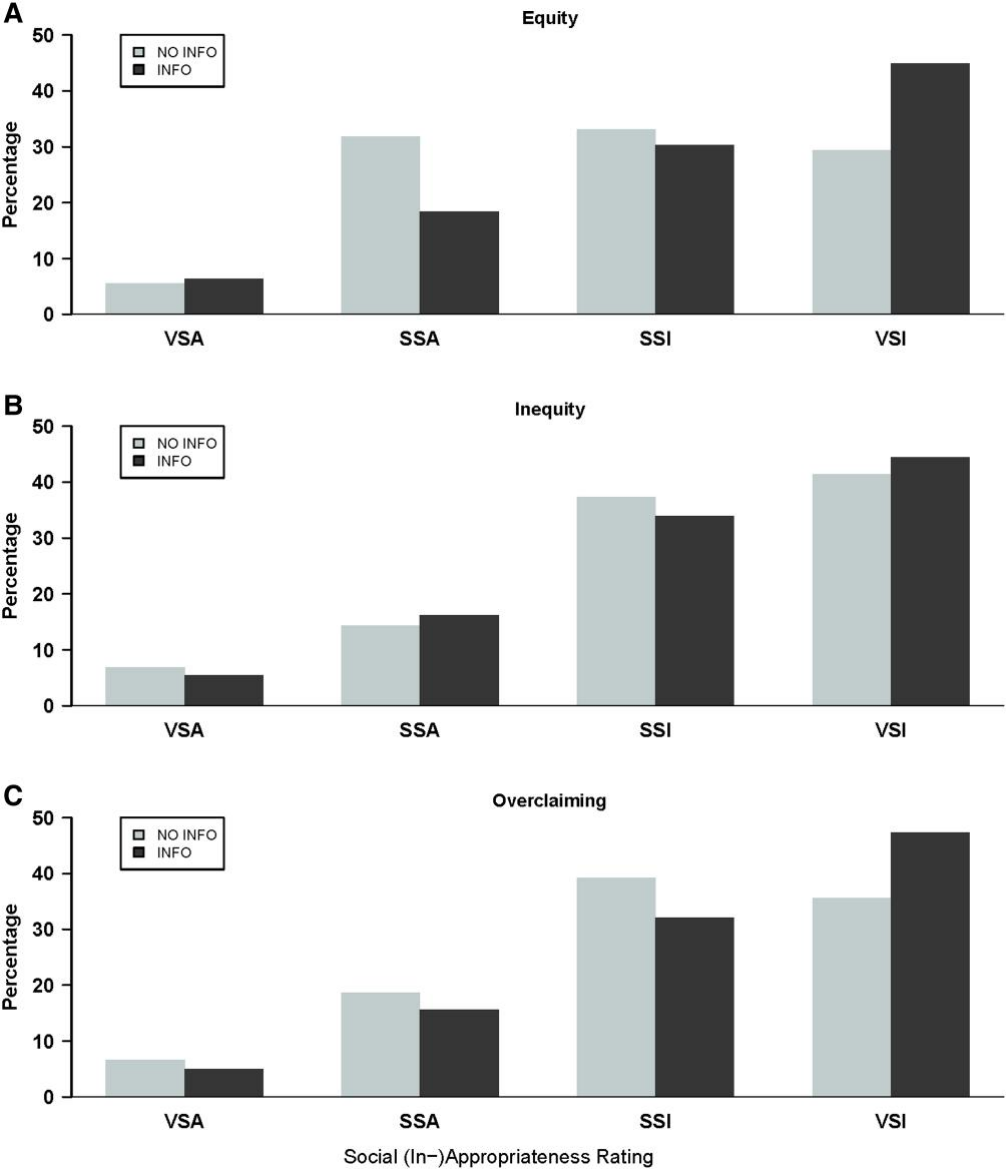
Social appropriateness of lying over different norm information and equity situations. A) Equity. B) Inequity. C) Overclaiming. *Note:* Each panel shows the distribution of responses for NO INFO and INFO treatments, always with an equity nature of the lie. INFO pools the NORMATIVE and EMPIRICAL treatments. The social norm is measured via a 4-item Likert scale ranging from “very socially appropriate” (*VSA*) over “somewhat socially appropriate” (*SSA*) and “somewhat socially inappropriate” (*SSI*) to “very socially inappropriate” (*VSI*).

Fig. 5 illustrates the distributions of social (in)appropriateness ratings, split by whether norm information is provided or not over different equity natures of the lie. We first examine the role of the (in)equity-scenarios, then the role of norm information, and lastly their interaction and relate this to that figure.

#### Norm information

We find a similar effect for the norm-nudge as in the behavioral experiment. In particular, we do not see a difference in responses between the two norm information treatments NORMATIVE and EMPIRICAL (rank-sum test: P=0.198). Consequently, we pool these two scenarios for the remainder of the analysis.^n^ When we compare the effect of norm info across the different equity scenarios, we find that providing norm information (INFO) leads to a lower average social appropriateness rating of lying (rank-sum test: NO INFO vs. INFO, P<0.001). In Fig. 5 this becomes evident by the shift of mass to the right, towards more socially inappropriate rating, when comparing the NO INFO-distribution to the INFO-distributions.

#### Equity nature of the lie

Aggregated over the norm-nudge information treatments, the average rating in the Inequity- and Overclaiming-scenarios do not differ but indicate a higher social inappropriateness than in the Equity-scenario (visualized by less combined mass on the right in Panel A vs. Panels B and C in in Fig. 5). This pattern is also marginally significant, according to pairwise rank-sum tests (Equity vs. Inequity P=0.063, Equity vs. Overclaiming P<0.066, Inequity vs. Overclaiming P=0.983; all rank-sum test reported here and in the following are two-sided). This shows that the perception of social norms across different equity settings reflects the punishment patterns observed in the behavioral experiment of Study 1.

#### Interaction of norm information and equity nature

For the NO INFO treatments, the perceived normative appropriateness is relatively heterogeneous across the various equity-scenarios. Panel A in 5) shows that in the Equity-scenario, the perceived social norm towards lying in NO INFO is relatively forgiving, with *SSA*, *SSI*, and *VSI* each obtaining about one-third of the total ratings. In contrast, most subjects with NO INFO in the Inequity-scenario (Panel B) assess lying to be “very socially inappropriate” (*VSI*). For the Overclaim-scenario (Panel C), the pattern is similar. In line with this, the NO INFO responses in the Inequity- and Overclaiming-scenarios do not differ significantly (rank-sum test: P=0.261). In contrast, NO INFO responses in the Equity-scenario differ significantly from responses in the Inequity- and Overclaim-scenarios (rank-sum tests: P=0.003 and P=0.061, respectively).

These patterns differ when norm-INFO is provided. The dark-colored distributions of perceived social appropriateness are the same, irrespective of the lie’s equity nature (i.e. across all panels of Fig. 5): *VSI*) is always the modal answer and across all equity-conditions, only a small share of subjects considers lying to be appropriate. This homogeneous pattern induced by norm information across equity conditions is also reflected in rank-sum tests which do not indicate any significant differences (P≥0.311 for all pairwise comparisons of the responses among the three treatments with norm-INFO and different equity scenarios).

Note that the provision of norm information helps people coordinate: in the INFO treatments, the frequency of the modal category *VSI* is always substantial. In the NO INFO treatments, this coordination on the modal category is overall less pronounced. If we examine this effect of norm information conditional on the equity scenario, we see that norm-nudges are effective when there is a conflicting social norm such as when the liar is initially disadvantaged and lying can restore equity (i.e. in the Equity-scenario). This manifests in the observation that norm-INFO leads lying to be perceived as more socially inappropriate relative to the NO INFO treatment Equity-scenario (comparison of distributions in Panel A, rank-sum test: P<0.001), as well as in the Overclaiming-scenario (comparison in Panel C, rank-sum test: P=0.020). In contrast, we do not find a significant difference in the case of Inequity-achieving lying, where the preexisting norm against lying was already relatively strong (comparison in Panel B, rank-sum test: P=0.645). These findings are also supported by regression analysis in Table S6 in online supplementary material A. It shows a concentration of the effect of norm info on appropriateness ratings in the Equity-scenario and no effects in the Inequity- and Overclaiming-scenarios.

## Discussion

In Study 1, we explore the drivers of norm enforcement in the context of lying. Participants observe liars with varying degrees of dishonesty and equity consequences across different norm information settings. We find that punishment is higher for larger lies and lies that increase inequity for the liar, while punishment is less severe when the lie offsets an ex-ante imbalance. These results are confirmed in our vignette experiment (Study 2).^o^

Study 3 investigates *why* norm enforcement varies across the settings in the first experiment. Using a separate online sample, we elicit social norm perceptions across the same lying settings. Our results show that inequity-based lies are perceived as less acceptable than equity-based lies, and providing norm information leads to increased punishment, regardless of the information’s empirical or normative nature. This is also reflected in Study 3’s elicited norm perceptions, suggesting a close link between variations in punishment and norm perception.^p^ Our experiments demonstrate that norm-nudges, such as providing norm information, can foster norm enforcement. A deeper analysis reveals that the impact is driven by the shift in norm perceptions: In Study 1, we find that the overall positive effect of norm information on punishment is weaker in the Overclaiming-scenarios as compared to the Inequity- and Equity-scenarios (see online supplementary Table S4 and its discussion). Conversely, Study 3 also shows that norm-nudges are less effective in situations where clear norms exist but are not necessarily honored. Together, we find particularly strong effects of norm info on both—punishment behaviors*and* norm perceptions—for Equity-based lies, while this combined pattern is relatively weak for Overclaiming-scenarios (with mixed results for Inequity-scenarios). Consistent with Merguei et al. (65), our interpretation is that norm-nudges work by reducing normative uncertainty for punishers.^q^

It is important to note that our experimental design and the results that come from it can capture key considerations and consequences for norm enforcers and violators in applied settings. In practice, social norms are often not clear-cut and are in conflict with other norms, similar to lying in our inequity-scenarios. Furthermore, even though punishment in natural contexts is often nonpecuniary (e.g, in the form of disapproving gazes and shaming; see Refs. (66, 67)), it often comes with reputational and pecuniary costs (e.g. from becoming socially ostracized or being excluded from cooperative interactions; see Bolton et al. (68)). In fact, Masclet et al. (69) show that monetary and nonmonetary enforcement are comparable in their effectiveness for sustaining cooperation. Given that our key experimental results in the context of the stylized framing replicated in the arguably more applied framing of the vignette, we are therefore optimistic that the findings from our experimental setup can yield valuable insights for the design and evaluation of “real-world” policies and norm-nudges.

## Conclusion

The enforcement of social norms is crucial for maintaining a functioning society, as unpunished transgressions can undermine this foundation (27). Although enforcing norms can be individually costly, it can lead to substantial collective gains by promoting coordination and norm adherence (see Xiao (70) for a recent review). While much of the existing literature has focused on the *effect* of punishment in social interactions and its ability to uphold social norms, less attention has been given to the *drivers* of such punishment. Understanding the circumstances under which observed norm transgressions are punished and the motives behind these transgressions are essential. Our contribution emphasizes key aspects of norm enforcement: its multilayered nature, the influence of norm-nudges, and its connection to norm perception.

We investigate the sensitivity of norm enforcement to the consequences of observed transgressions (e.g. achieving equity vs. obtaining an unfair advantage) and the type of norm-nudge (empirical vs. normative). Our analysis is conducted through three experiments: the first two focus on measuring norm-*enforcement* behavior, while the third targets the associated norm-*perceptions* of the observed lies. This enables us to not only understand how norm transgressions are punished but also determine the extent to which variations in norm enforcement correspond with variations in norm perceptions. Our findings indicate a strong alignment of both aspects.

With that, the findings from our various empirical approaches complement each other in a contribution that goes beyond the existing literature. Specifically, we provide a parsimonious

1. examination of how norm enforcement responds to norm-nudging (*Study 1*),
2. verification that this effect is not limited to the behavioral game that we test but is also mirrored in our vignette experiment examining deviance in a business context (*Study 2*),
3. indication for the mechanism of the observed behavior in that the norm-nudges seemingly provide a corresponding shift in norm perceptions (*Study 3*).

Our “modular approach,” utilizing various experimental methods, is well suited for obtaining policy-relevant insights (71–73). From a policy standpoint, our results underscore the importance of considering the diverse social motivations of both transgressors and norm enforcers, particularly when employing “soft” interventions like nudges for behavioral change. Previous research indicates that norm-based interventions, such as norm-nudges, must be carefully tailored to the social environment in which they are implemented. Our findings contribute to the ongoing scholarly discussion on why earlier studies utilizing soft norm-nudge interventions have encountered mixed success (16, 63, 74, 75). For instance, we observe that norm-nudges have the greatest impact when preexisting norm perceptions are relatively unclear (see Footnote 17 discussion). This aligns with research demonstrating that even when individuals recognize a norm, ambiguous norm perceptions can undermine compliance, as they can be exploited for self-serving purposes (37, 76). In an applied context, and consistent with the libertarian paternalism approach (9), policy-makers can implement the nudge interventions tested here by strategically providing relevant social information to those with norm enforcement power. Given the observed responsiveness of norm enforcers to such interventions, our study offers an additional dimension for choice architects to effect behavioral change (77).

Existing research on nudging has predominantly adopted an *individualistic* perspective, focusing on facilitating behavior change by directly targeting individuals. While this approach can be successful, the immediate effectiveness and longevity of such interventions vary considerably (13, 78, 79). In contrast, we explore the *collectivist* perspective on behavior change by targeting those who enforce behavior rather than those whose behavior is intended to be altered. Peer mechanisms can effectively uphold norms, even in the absence of formal rules and laws. Our findings offer an additional perspective on overcoming nudging challenges (72, 80, 81). By supplementing the arsenal of behavioral change techniques targeting individual decision-making (streamlining decision environments, defaults, etc.), policy-makers can generate momentum at the collective level by focusing on those who can enforce norm adherence. As Bujold et al. (82) argues, successfully implementing this form of “meta-nudging” is particularly important for policy-makers in various contexts. This strategy may be fruitful in the context of social movements, where social change can be facilitated by social influencers (83). In such cases, using meta-nudging to trigger a *change cascade* can be particularly promising (84). More specifically, meta-nudging can “supercharge” classical nudging approaches by increasing norm adherence (85). To achieve this, meta-nudging must target the right peers, called *social influencers*, who then delegate the policing of existing norms in the form of “hired guns.” When nudged in the right direction, these individuals can become *social trendsetters* willing to bear the cost of initiating change due to their typically lower risk sensitivity (27). A potential advantage of meta-nudging is that behavioral interventions relying on delegated policing might be perceived as less intrusive and more successful, capitalizing on existing peer mechanisms (86). This could arguably increase the acceptability of enforcement, a critical component of successful norm enforcement (3). Our experiments suggest that individuals can be successfully meta-nudged, leveraging the social impact of peers to effect behavior change.

The recent scholarly debate (e.g. Maier et al. (87)) has questioned the uniform effectiveness of nudges on behavior change, arguing that nudges often have small effect sizes and that publication bias may attenuate documented effects. However, as other scholars convincingly argue (e.g. Refs. (88–90)), it is essential to consider the heterogeneity of nudging with respect to intervention, setting, and populations when evaluating effectiveness. Various meta-studies (e.g. Refs. (10, 13, 79)) have found that norm-nudges (as tested here) are among the most effective nudge interventions. As our studies were preregistered, well-powered, and utilized diverse subject pools and designs, we are confident in our findings’ validity.

Our work advances this scholarly debate by identifying effective nudging strategies and examining the reasons for unsuccessful nudging. Our results indicate that norm enforcers readily use punishment sensitive to the motives underlying a norm breach, suggesting a “hands-off” approach can be justified when norm adherence is self-enforcing through peer punishment. However, we find that simple norm-nudges do not necessarily change norm perception or punishment extent for firmly established norms. To strengthen and sustain norm enforcement, norms within peer groups should be reinforced through messages raising awareness for applicable norms. Combined with opportunities to punish, as in our experimental setting, this strategy may facilitate enforcement of norm compliance when peer conformity alone is insufficient.

To achieve behavior change and foster norm enforcement, it is crucial to understand the context and existing norms of the intended behavior. This may involve relying on gentle norm-nudges as studied here, stronger interventions such as *shoves* and *boosts*, or even explicit economic incentives (91). Evaluating the boundary conditions under which the enforcement nudges tested here will work aligns with the recent call for a more comprehensive evaluation of nudges’ heterogeneous effects (92). Our research highlights the importance of adopting a collective perspective on nudging, an underutilized aspect in existing theoretical and applied work, and encourages future policy efforts in this direction.

## Supplementary Material

pgad224_Supplementary_Data

## Data Availability

The code and replication materials used for this study are available on OSF at https://osf.io/nfkyc.

## Notes

a See, e.g. Ostrom (34), Kimbrough and Vostroknutov (93), Albrecht et al. (94), Fehr and Schurtenberger (95), Schurtenberger (96), Choi et al. (57), Bolton et al. (52), Galeotti et al. (97), Dimant (98), and Bicchieri et al. (99).

b For applications see, e.g. Hallsworth et al. (75), Allcott and Kessler (100), Bursztyn et al. (101), and Bursztyn et al. (62).

c To meet the criteria for robustness and generalizability of MTurk findings (e.g. Refs. (102, 103)), we applied high-quality restrictions on the sample: We used a combination of CAPTCHAs and comprehensive screening questions. Participants had to be in the US and have an approval rate >99%.

d Data collection was conducted through the market research firm Dynata (formerly “Research Now”) using a quota-based sample, with the aim of having representativeness for age and gender based on the US population.

e To ensure the validity of this information, we conducted the auxiliary experiment for EMPIRICAL with n=52 subjects who were in the same decision situation as reporters in the Liar Experiment, except that they could not revise their statement since there was no punishment. The majority of them (57.7%) reported truthfully. The auxiliary experiment for the NORMATIVE treatment involved n=51 subjects who were told about the Liar Game and the reporter’s decision scenario. They then had to report what they considered to be “the right thing to do.” The majority (68.6%) chose that reporting the actual outcome (i.e. to tell the truth) was the right thing to do.

f For example, if such a player rolled a “2” but reported a “4” and the matched punisher assigned x∈{0,…,5} punishment points for this scenario, that player’s earnings would be reduced by *x* × $0.3. Note that the relevant report for punishment was the second, potentially revised, report submitted by the active player. Also, while punishment affected that player’s earnings if matched with the punisher, it did not affect the lottery tickets and chances to win the bonus.

g We also did not allow the punishment of downward lying because i) it is not relevant for actual liar behavior (see our Results section), ii) it is not pertinent to our main research question on (conflicting) norms and norm transgressions, and iii) it would have doubled—and thereby complicated—the action space of punishers, diverting their attention and deliberation from the scenarios of interest.

h The matching ratio of punishers to active players was roughly 15:1. The instructions did not state or suggest any explicit value for this ratio but our exit questionnaire for punishers asked to provide an estimate for it. The average value (excluding one extreme outlier) implies a ratio of 1:0.870—punishers, therefore, overperceived the consequences of their decision. Also, these beliefs did not differ significantly between treatments (implied ratios of 0.881, 0.925, 0.806 in NO INFO, NORMATIV, EMPIRICAL, respectively; Kruskal-Wallis-test: P=0.548). We also control for these beliefs in our regression analysis, without finding it to influence results (see online supplementary material A).

i Liars received an average payment of $2.75, they spent on average 12:29 min in the experiment (passive players received an average $2.51 over 13:18 min). We paid the survey company Dynata €2 per punisher (≈$2.25 at the time of the experiment); their time in the experiment was on average 14:32 min.

j For each size of the lie, one can form pairs of punishment scenarios where the lie increases inequity as much as it decreases it: p45/p12, p34/p23, p25/p14, and p35/p13. For the remaining pair p15/p24, the inequity in the chances to obtain the bonus remains constant but flips who benefits from this inequity. Note that punishment can also not be used to decrease (expected) inequity via the punishment amount which is subtracted from the lying player’s earnings. The reason is that the size of the lie and inequity are uncorrelated: higher punishment for larger lies then only increases (expected) inequity.

k The study duration was approximately 5 min, and participants had a chance to win one of five $100 vouchers via a random lottery for their participation.

l Following norms literature conventions (e.g. Refs. (63, 98, 104)), we elicit norm beliefs from participants who did not take part in Study 1, avoiding potential issues like priming, demand effects, or post hoc justifications by subjects (see d’Adda et al. (105) for a discussion).

m For a breakdown of observations per treatment see Table S5 in the online supplementary material where we also describe subject characteristics and provide randomization checks. The average duration of the experiment was about four minutes. Participants were paid a show-up fee of $0.20 with an opportunity to receive a $0.20 bonus based on their answers and thus were paid well above average MTurk pay (106). We used the same participant pool restrictions used in the behavioral experiment of Study 1 (see Endnote 3).

n Specifically, we find that appropriateness ratings in EMPIRICAL and NORMATIVE are significantly higher than ratings in NO INFO (rank-sum tests: EMPIRICAL vs. NO INFO, P=0.012; NORMATIVE vs. NO INFO, P<0.001). Note that the norm info in the NORMATIVE treatment is somewhat closer to what we elicit (subjects’ perceived social norm) than the info in EMPIRICAL. This is why we also provide in online supplementary Fig. S5 and further tests in online supplementary material A where these treatments are not pooled; the results are largely the same. Also note that online supplementary Fig. S4 shows a related figure, with a similiar effect of norm info for the punishment data from Experiment 1.

o Our results also relate to existing research examining how equity concerns, social perception, and the size of a lie affect dishonest behavior (see, e.g. Refs. (49–51, 56, 107)). This literature generally focuses on the factors determining the *intrinsic* costs of lying, whereas we examine how these factors shape the *extrinsic* costs through punishment.

p Recall that the behavioral experiment in Study 1 is a within-subjects design with respect to the different lying scenarios, whereas Study 3 varies all treatment dimensions between subjects. Importantly, the fact that the results are very consistent in these independent experiments suggests that our results are robust to both of these design choices (see also Clifford et al. (108) for a recent discussion on the robustness of within-designs).

q This is consistent with findings by Goldstein et al. (59), who investigated the impact of norm information provision on environmentally friendly behavior (towel reuse in hotels). Importantly, however, a conceptual replication by Bohner and Schlüter (109) failed to replicate this in Germany. The authors argue that this is likely due to the already existing strong norm of environmental preservation. Additional information was not informative and therefore did not produce any behavior change (see Dimant et al. (63) for related findings on lying). Note that, in principle, this interpretation is also consistent with the notion of pluralistic ignorance, where individuals erroneously perceive their thoughts, feelings, or behaviors as divergent from the majority, despite actual similarities (110). While we lack a direct method to test this, as participants’ norm beliefs were not assessed prior to the intervention, the tested norm-nudges could potentially improve norm enforcement by rectifying pluralistic ignorance and refining norm beliefs accordingly. If so, this would be consistent with our results that norm enforcement increases following our norm-nudging.

## Preprints

This manuscript was posted on a preprint: https://dx.doi.org/10.2139/ssrn.3664995.
