# Supplementary material for: Nudging enforcers: how norm perceptions and motives for lying shape sanctions
Source: PNAS Nexus. 2023 Jul 4;2(7):pgad224. doi: 10.1093/pnasnexus/pgad224 (PMC10360164; doi:10.1093/pnasnexus/pgad224)
Supplement: pgad224_Supplementary_Data [file pgad224_supplementary_data.pdf]

Nudging Enforcers: How Norm Perceptions and Motives for Lying Shape Sanctions

Supplementary Material

Contents:

A: Additional figures and data analysis

B: Details on the Vignette Experiment in Study 2

C: Information regarding pre-registration

D: Instructions

1. Instructions for the Liar Experiment in Study 1
2. Instructions for the Behavioral Experiment (Punishers) in Study 1
3. Instructions for the Vignette Experiment in Study 2
4. Instructions for the Norm Elicitation Experiment in Study 3

Appendix A: Additional figures, tables, and data analysis

Additional analyses for Study 1

Figure S1 provides a systematic breakdown of a **liar's** behavior (both first report and revised report) conditional on the random outcome of the die toss. We can observe a consistent revision pattern in that liars are less likely to misreport the number after being made aware of the punishment risk.

Figure S1: Liars' first and revised reports by the actual result of the die

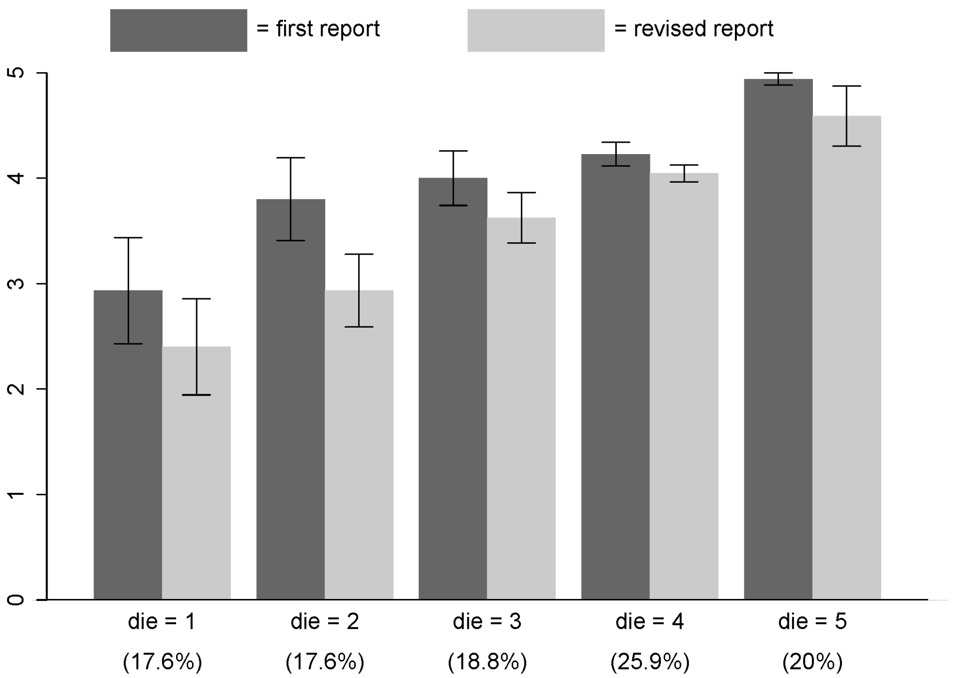

Notes: Means of first and revised reports for the outcome of the 5-sided die toss, grouped by the actual outcome of the die toss (frequency for each outcome in parentheses). Error bars denote SEM.

Figure S2 a systematic breakdown of a **punisher's** behavior conditional on the punishment scenario and the size of the lie, as illustrated in Figure S1. We observe a clear pattern in that the share of assigned punishment increases with the size of the lie.

Figure S2: Punishment for each punishment scenario, ordered by size of the lie

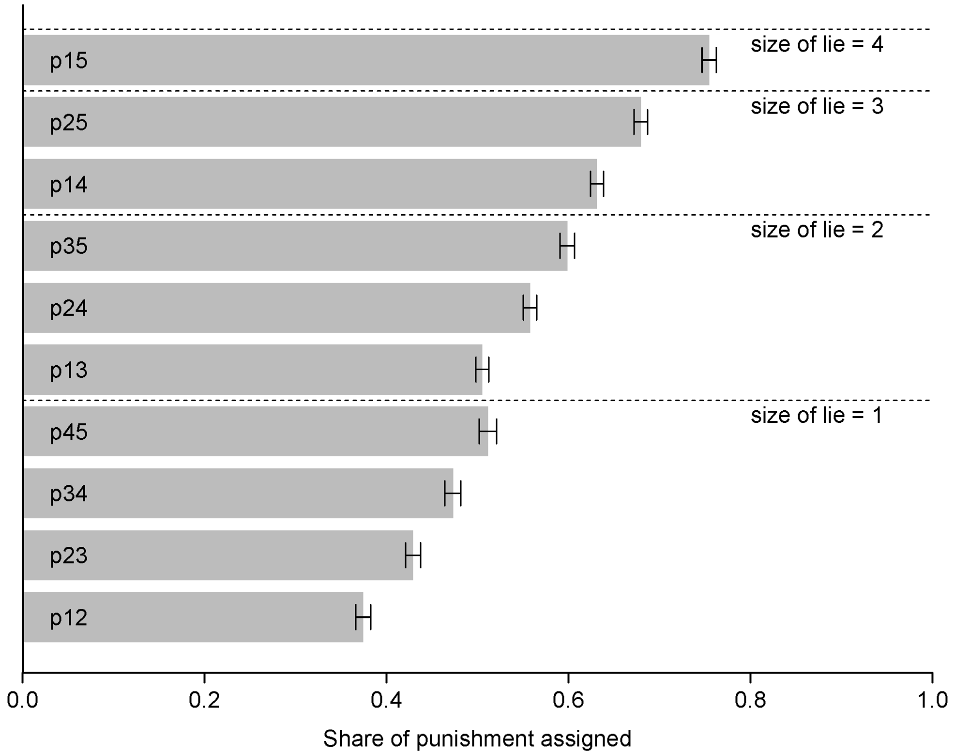

Notes: Punishment is assigned as a share of total punishment points available in each punishment scenario, ordered by the size of the lie. Error bars denote SEM.

Figure S4 illustrates the results from Figure S2 in a different way. In particular, the figure below breaks down the share of assigned punishment conditional on the equity nature of the lie. The results suggest that lying to overclaim (achieve equality) is punished the most (least), which is consistent with our results from Study 3.

Figure S3: Punishment for each punishment scenario, ordered by equity norm

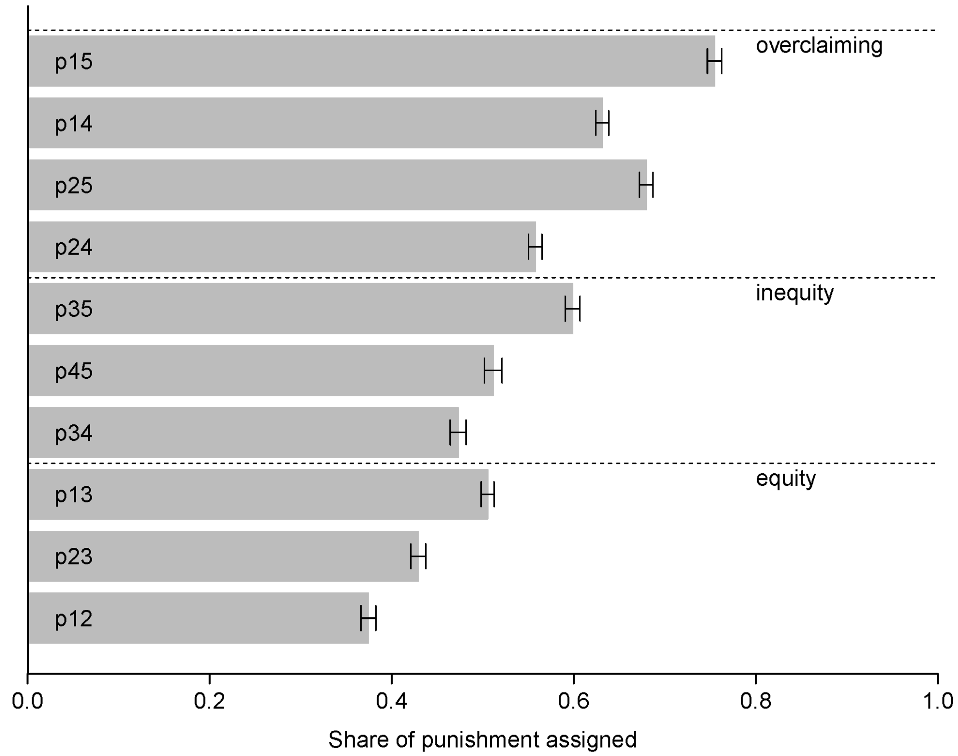

Notes: Punishment is assigned as a share of total punishment points available in each punishment scenario, ordered by the equity nature of the lie. Error bars denote SEM.

Figure S4: Distribution of punishment decisions over info treatments in P13, P35, and P24

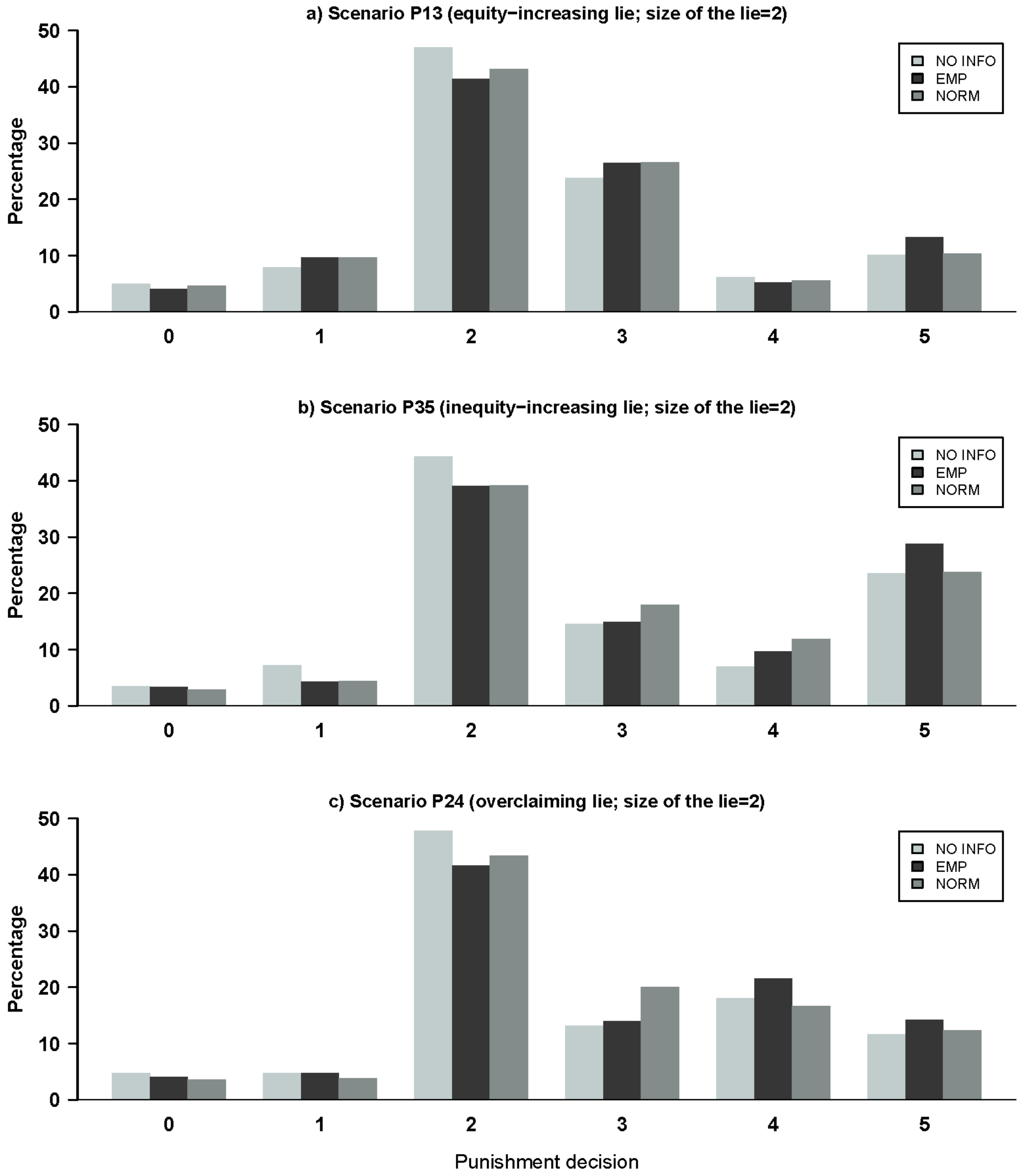

Notes: Each panel shows the distribution of punishment for a (within-person) equity scenario over different norm information treatments. All panels differ in the inequity nature of the lie but keep the size of the lie constant.

Table S1: Descriptive statistics for active players

	Mean	S.D.
Age	36.835	10.194
Male	0.682	0.468
Edu.: some high school	0.012	0.108
Edu.: finished high school	0.118	0.324
Edu.: some college	0.341	0.477
Edu.: finished college	0.447	0.500
Edu.: higher degree	0.082	0.277
observations	N=	85

Notes: Personal characteristics of active players in the pre-experiment (mean and standard deviation).

Table S2: Descriptive Statistics for punishers by norm information treatments

	NO INFO		NORMATIVE		EMPIRICAL	
	Mean	S.D.	Mean	S.D.	Mean	S.D.
Age	43.545	13.478	42.552	14.122	43.303	14.089
Male	0.468	0.500	0.477	0.500	0.482	0.500
Edu.: some high school	0.037	0.189	0.039	0.193	0.024	0.152
Edu.: finished high school	0.163	0.370	0.245	0.430	0.194	0.396
Edu.: some college	0.280	0.449	0.257	0.437	0.277	0.448
Edu.: finished college	0.300	0.459	0.293	0.456	0.314	0.465
Edu.: higher degree	0.208	0.406	0.160	0.367	0.175	0.380
observations	N=	404	N=	413	N=	423

Notes: Demographic characteristics of punishers in Study 1, by norm-information treatment (mean and standard deviation). We do not observe statistically significant differences between the treatments for age (Kruskal-Wallis test: p=0.548), gender (χ^2-test: p=0.916), or education (χ^2-test: p=0.190).

Regression analysis: In addition to our non-parametric results, we also examine our data by fitting the following regression equation

$$\begin{aligned} p_{is} = \alpha \quad & +\beta_1 \text{ EMPIRICAL}_i + \beta_2 \text{ NORMATIVE}_i \\ & +\beta_3 \text{ LieSize}_s + \beta_4 \text{ Inequity}_s + \beta_5 \text{ Overclaiming}_s + \boldsymbol{\gamma'} \textbf{ Controls}_\mathbf{i} + \epsilon_{is}. \end{aligned} \tag{1}$$

In the regression above, the dependent variable p_{is} is the share of punishment by subject i in the scenario s, expressed as a number between 0 and 100. Consequently, the coefficients for the independent variables can be interpreted as percentage-point shifts. The independent variables in (1) are the following: EMPIRICAL_i and NORMATIVE_i are dummies indicating whether subject i was in one of the norm nudge treatments (or in NO INFO, the baseline category), $LieSize_s$ measures the size of the lie in punishment scenario s, while Inequity_s and Overclaiming_s are dummies indicating the equity norm of scenario s (with an Equity-scenario being the baseline). Age, gender, and education are collected in the $\textbf{Controls}_\mathbf{i}$-vector for each punisher. We then fit the above model using OLS with standard errors clustered at the subject level i.

Table S3 reports the regression results. The first three columns repeat our previous statistical analysis parametrically. In Column (1), we replicate the finding that larger lies lead to significantly more punishment by about 10 percentage points for each unit increase in the size of the lie. Column (2) shows that relative to Equity-based punishment scenarios, Inequity- and Overclaim-scenarios lead to significant increases in the share of punishment assigned by about 9 and 22 percentage points, respectively. We also find that the implied difference of 13 (=22−9) percentage points between the Inequity- and Overclaim-scenarios is statistically significant (F-test: $p<0.001$). Column (3) shows that providing empirical and normative information leads to a significant increase in punishment of about 2.7 percentage points (significant at the 10% level) and 3.5 percentage points (significant at the 5% level), respectively. These coefficients do not differ significantly from each other (F-test: $p=0.629$)

The full model in Column (4) allows us to measure the effect of different equity scenarios while controlling for differing lie sizes in the associated scenarios where the underlying lies differ in motive (i.e., in the Overclaiming-scenarios). This is particularly important in order to check the robustness of the results concerning Equity-norms in Column (2), as these results could also be due to the differing sizes of lies in the Inequity- and Overclaim-scenarios. Consistent with this caveat, we find that the coefficient for the Overclaim-scenarios shrinks by 17.1 punishment points (Column 2 vs. 4). This number corresponds very closely to the difference between the average size of the lie across the four Overclaim scenarios (p14, p15, p25, and p24; average lie size=3) minus to the average across the baseline Equity scenarios (p12, p13, p23; average lie size=1.3), multiplied with the "Size of the Lie"-coefficient of 10.1. Thus, these results are internally consistent. However, even when controlling for the larger lie sizes, the Overclaim-scenarios remain significantly positive, with a 5.1 punishment points increase relative to the baseline Equity-scenarios. Thus, while smaller than initially suggested, we continue to find

Table S3: Determinants of punishment – regression results

	Share of punishment assigned in %			
	(1)	(2)	(3)	(4)
Size of the Lie	10.321***			10.110***
	(0.295)			(0.324)
Inequity		9.140***		9.140***
		(0.532)		(0.532)
Overclaiminig		21.948***		5.098***
		(0.523)		(0.402)
EMPIRICAL			3.469**	3.469**
			(1.648)	(1.648)
NORMATIVE			2.695*	2.695*
			(1.607)	(1.607)
Constant	31.604***	40.725***	50.055***	25.055***
	(2.741)	(2.692)	(2.845)	(2.879)
N	12,400	12,400	12,400	12,400
R^2	0.123	0.099	0.008	0.139

F-tests for coefficients on:
- Inequity = Overclaiming: $p<0.001$
- EMPIRICAL = NORMATIVE: $p=0.628$ (in both, columns 3 and 4)

Notes: OLS results regressing the share of punishment assigned on the size of the lie, the equity nature of the lie (inequity, overclaiming), and indicators for the norm information treatment (EMPIRICAL or NORMATIVE); the baseline category is a subject in treatment NO INFO and an equity-based punishment scenario with lie size=1. Additional control variables include age, gender, and education. 10 punishment scenarios per punisher; standard errors are clustered at the punisher level. */**/***: p<0.10/0.05/0.01.

support for Hypothesis 2b: (In)Equity concerns do not only matter when lying creates inequality but also when it reverts pre-existing inequality that benefits the liar. Overall, the regression results (re-)confirm Hypotheses 1, 2, and 3.

Table S4 (next page) follows regression equation (1). Instead of using the EMPIRICAL$_i$- and NORMATIVE$_i$-dummies, however, it uses a an INFO$_i$-dummy, with different meanings across three specifications covering different data:

1. Only data from treatments the baseline NO INFO (INFO$_i = 0$) and EMPIRICAL (INFO$_i =$ 1).

2. Only data from treatments the baseline NO INFO (INFO$_i = 0$) and NORMATIVE (INFO$_i = 1$).

3. Data from treatments NO INFO (INFO$_i = 0$) and both NORMATIVE and EMPIRICAL (INFO$_i = 1$); the latter two treatments are therefore pooled.

This also features interactions of the INFO$_i$-dummy and the Inequity$_i$- and Overclaiming$_i$-dummies. The three columns in Table S4 correspond to the three specifications above and show no qualitative differences in the results and the test of our hypotheses.

Finally and in addition to the above specifications (where in the case of Table S3 this represents the pre-registered regression tests) we also repeat the above regressions checks with an appended $\mathbf{Controls_i}$-vector. Specifically, we add a dummy to control for the order in which scenarios were presented (counterbalanced over treatments). Furthermore, we add punisher i's estimate for the ratio of punishers to reporters in Part 1 (that is, the implementation probability of punishment; we never find this to be significant). We also performed additional checks by adding punisher fixed effects in the specifications which only feature within-subject variations (i.e., those in columns 1 and 2 of Table S3; the between-subject norm INFO-variables in the other specifications get absorbed by the fixed effects). None of these appended regressions changes any of our results.

Table S4: Differential effect of norm info on punishment – regression results

	Share of punishment assigned in %		
	(1)	(2)	(3)
INFO	3.668**	2.915*	3.583**
	(1.780)	(1.753)	(1.526)
Inequity	8.168***	8.168***	8.168***
	(1.146)	(0.867)	(0.867)
Overclaim	6.247	6.344***	6.802***
	(0.791)	(0.783)	(0.811)
INFO × Inequity	1.067	1.824	1.441
	(1.062)	(1.283)	(1.094)
INFO × Overclaiming	-2.749*	-2.299*	-2.527**
	(1.498)	(1.254)	(1.073)
Lie	10.110***	10.384***	10.110***
	(0.324)	(0.393)	(0.324)
Constant	24.649***	20.552***	19.640***
	(2.891)	(3.694)	(3.089)
INFO refers to	EMPIRICAL ONLY	NORMATIVE ONLY	EMPIRICAL & NORMATIVE
N	8,270	8,170	12,400
R^2	0.145	0.148	0.139

Notes: OLS results regressing the share of punishment assigned on the size of the lie, the equity nature of the lie (inequity, overclaiming), a dummy for whether norm information was provided (INFO) and interactions of the latter with the equity nature; the baseline category is therefore a subject in treatment NO INFO and an equity-based punishment scenario with lie size=1. INFO in column 1/2 designates treatment EMPIRICAL/NORMATIVE, respectively as opposed to NO INFO (no data from treatment NORMATIVE/EMPIRICAL used, respectively); in column 3 INFO designates treatments EMPIRICAL and NORMATIVE (all data used). Additional control variables include age, gender, and education. Robust standard errors in parentheses. */**/***: p<0.10/0.05/0.01.

Additional analyses for Study 3

Figure S5 is a more fine-grained breakdown of Figure 5 in that the norm conditions are also broken down by normative and empirical information. Because both norm conditions yield consistent results, we collapsed them under 'Information' in Figure 5 in the main text. This corresponds also to what we pre-registered (see Appendix C).

Together with Figure S5, we also report here the main comparison when done over the unpooled info treatments NORMATIVE AND EMPIRICAL. Note that for the first comparison ("Norm information"), this is already presented in Footnote 14. For the next comparison ("Equity nature of the lie"), this pools across all info treatments. Thus, the remainder is in the main analysis of the interaction of different equity-scenarios and norm info ("Interaction of norm information and equity nature"). The below reports this, all tests are ranksum test:

Norm-info effects in Equity-scenarios:

- EMPIRICAL vs NO INFO: p=0.018
- NORMATIVE vs NO INFO: p<0.001
- EMPIRICAL vs NORMATIVE: p=0.182

⇒ in the Equity-scenario, both norm info treatments (EMPIRICAL or NORMATIVE) leads to any significant change in social (dis-)approval ratings for lying compared to NO INFO. These two INFO treatments do not differ significantly from each other.

Norm-info effects in Inquity-scenarios:

- EMPIRICAL vs NO INFO: p=0.697
- NORMATIVE vs NO INFO: p=0.634
- EMPIRICAL vs NORMATIVE: p=0.949

⇒ in the Inequity-scenario, neither norm info treatment (EMPIRICAL or NORMATIVE) leads to any significant change in social (dis-)approval ratings for lying compared to NO INFO. These two INFO treatments do not differ significantly from each other.

Norm-info effects in Overclaiming-scenarios:

- EMPIRICAL vs NO INFO: p=0.094
- NORMATIVE vs NO INFO: p=0.017
- EMPIRICAL vs NORMATIVE: p=0.379

⇒ in the Overclaiming-scenario, both norm info treatment (EMPIRICAL or NORMATIVE) lead to (marginally) significantly more social dis-approval rating for lying than no info. These two INFO treatments do not differ significantly from each other.

Figure S5: Social appropriateness of lying over different norm information and equity treatment

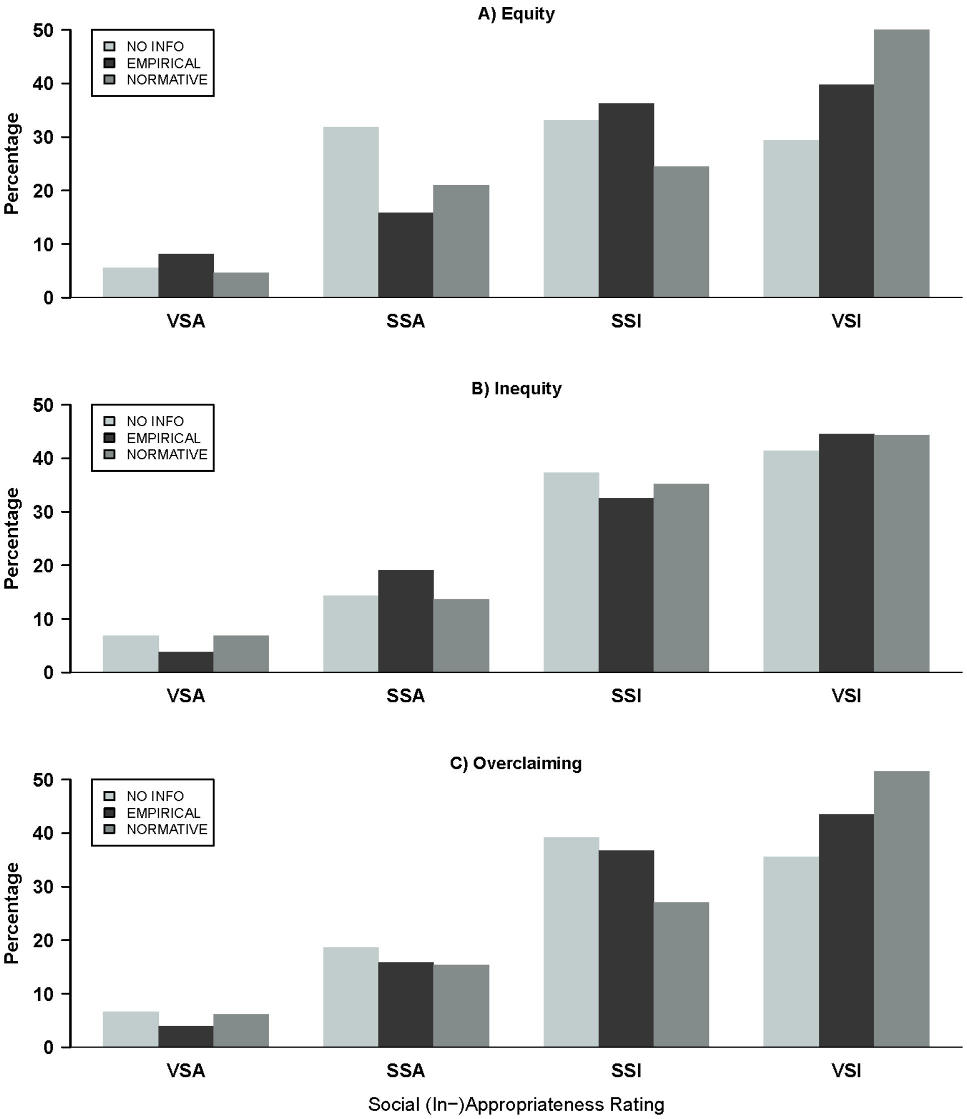

Notes: Each panel shows the distribution of responses for different norm information treatments, always with a different equity-nature of the lie. The social norm is measured via a 4-item Likert scale ranging from "very socially appropriate" (*VSA*) over"somewhat socially appropriate" (*SSA*) and "somewhat socially inappropriate" (*SSI*) to "very socially inappropriate" (*VSI*).

Table S5: Descriptive Statistics for subjects in Study 3

	Panel a) NO INFO					
	Equity		Inequity		Overclaiming	
	Mean	S.D.	Mean	S.D.	Mean	S.D.
Age	37.862	13.791	39.529	13.686	39.843	12.835
Male	0.469	0.501	0.483	0.501	0.506	0.501
Educ.: less than high school degree	0.000	0.000	0	0.000	0.000	0.000
Educ.: high school graduate	0.094	0.292	0.080	0.273	0.133	0.340
Educ.: some college but no degree	0.150	0.358	0.161	0.369	0.193	0.396
Educ.: associate degree in college	0.094	0.292	0.092	0.290	0.090	0.288
Educ.: bachelor degree in college	0.481	0.501	0.477	0.501	0.398	0.491
Educ.: master degree	0.131	0.339	0.172	0.379	0.157	0.365
Educ.: doctoral degree	0.025	0.157	0.006	0.076	0.006	0.078
Educ.: professional degree (JD, MD)	0.025	0.157	0.011	0.107	0.024	0.154
observations	N=	160	N=	174	N=	166

	Panel b) INFO					
	Equity		Inequity		Overclaiming	
	Mean	S.D.	Mean	S.D.	Mean	S.D.
Age	38.437	12.483	38.508	12.715	38.303	12.696
Male	0.469	0.500	0.456	0.499	0.435	0.497
Educ.: less than high school degree	0.012	0.108	0.000	0.000	0.003	0.054
Educ.: high school graduate	0.076	0.265	0.048	0.214	0.094	0.292
Educ.: some college but no degree	0.178	0.383	0.213	0.410	0.171	0.377
Educ.: associate degree in college	0.105	0.307	0.111	0.315	0.118	0.323
Educ.: bachelor degree in college	0.466	0.500	0.411	0.493	0.432	0.496
Educ.: master degree	0.128	0.335	0.180	0.385	0.144	0.352
Educ.: doctoral degree	0.012	0.108	0.012	0.109	0.026	0.161
Educ.: professional degree (JD, MD)	0.023	0.151	0.024	0.153	0.012	0.108
observations	N=	343	N=	333	N=	340

Notes: Demographic characteristics of subjects in Study 3 (mean and standard deviation), split by the different treatments (Equity norm: Equity, Inequity, and Overclaiming times NO INFO and INFO). We do not observe statistically significant differences between the six treatments for age (Kruskal-Wallis test: p=0.502), gender (χ^2-test: p=0.749), or education (χ^2-test: p=0.197). NO INFO pools the observations from treatments NORMATIVE and EMPIRICAL. If taken individually, the number of observations in NORMATIVE are n=172, n=176, and n=163 for the Equity-, Inequity-, and Overclaim-treatments, respectively; the corresponding number of observations in EMPIRICAL are n=171, n=157, and n=177.

Table S6: Social appropriateness ratings – regression results

	Social Appropriateness score (1 – 4)		
	(1)	(2)	(3)
INFO	-0.154^{***}		-0.261^{***}
	(0.050)		(0.088)
Inequity		-0.109^{*}	-0.268^{***}
		(0.058)	(0.100)
Overclaim		-0.109^{*}	-0.163
		(0.058)	(0.101)
INFO × Inequity			0.232^{*}
			(0.122)
INFO × Overclaiming			0.078
			(0.123)
Constant	1.348^{***}	1.215^{***}	1.472^{***}
	(0.186)	(0.176)	(0.195)
N	1,516	1,516	1,516

F-test: coeff. for INFO + INFO × Inequity = Inequity; p=0.140
F-test: coeff. for INFO + INFO × Overclaiming = Overclaiming; p=0.908

Notes: OLS results regressing the social appropriateness score (coded as "Very Socially Inappropriate"=1, "Somewhat Socially Inappropriate"=2, "Somewhat Socially Appropriate"=3, "Very Socially Appropriate"=4) on indicators for the norm information treatments (INFO – pools EMPIRICAL and NORMATIVE) and the equity nature of the lie (inequity, overclaim); the baseline category is therefore a subject in treatment NO INFO and an equity-based punishment scenario with lie size=1. Additional control variables include age, gender, and education. Robust standard errors are in parentheses. */**/***: p<0.10/0.05/0.01.

Appendix B: Details on the Vignette Experiment to corroborate the robustness of the results in the Behavioral Experiment of Study 1

Design

Our vignette featured a scenario where subjects imagined themselves in the role of an employee who observed a co-worker (Alex) in the same company taking a bribe amounting to $10,000 in order to favor one of the company's suppliers. The subjects could then blow the whistle and inflict punishment on Alex. Participants were able to choose from a range of potential whistleblowing actions (from not reporting at all, to reporting the case and also submitting tangible evidence). This then determined the probability of Alex being punished (from 0% to 100%, in 25 ppt. increments). Besides this feature, which reflects the staggered punishment in Study 1, two further crucial features of that behavioral experiment are also captured in this vignette experiment: i) whether Alex accepting the bribe creates EQUITY (the bribe was accepted to make up for not having received a bonus payment outside of Alex's control) or INEQUITY (bribe was accepted on top of a bonus payment), and ii) whether injunctive norm information (previous participants indicating that accepting a bribe is not the right thing to do) was present or not. We elicited the probability of punishing the co-worker twice for each subject in two *within-subject* treatments that were presented in random order:

- EQUITY-creating bribe treatment: Alex had been held accountable for a failed project tender. Even though Alex was not personally responsible for the failure, Alex was reprimanded by the company by being deprived of a $10,000 end-of-the-year bonus. We also established a payoff difference in order to capture the equity-aspect of the behavioral experiment. For this, the subject playing the vignette was told that she received the very same end-of-the-year bonus. By virtue of this setting, accepting the bribe – which also has a value of $10,000 – creates equity for Alex when compared with the co-worker's (i.e., the subject's) payoff.
- INEQUITY-creating bribe treatment: The setup was the same as in EQUITY, except that the failed tender did *not* result in Alex being reprimanded for it. Thus, both Alex and

Figure S6: Design of the Vignette Experiment in Study 1

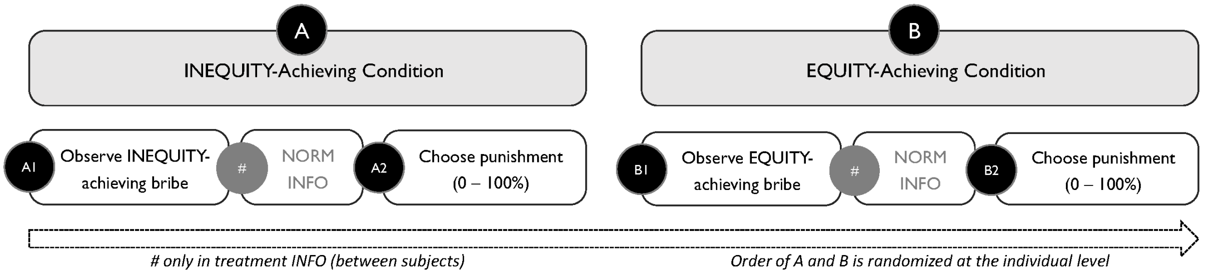

the subject received the $10,000 end-of-the-year bonus. Consequently, compared to the subject, accepting the bribe, therefore, creates an (advantageous) inequity for Alex.

In addition to the above, each subject was in one of two *between-subject* conditions:

- NORM INFO: In this treatment, subjects were presented with an empirical norm nudge similar to the one used in the behavioral experiment.[1] It informed them that in a previous study, the majority of participants agreed with the statement that "*taking a bribe is highly unethical and not the right thing to do.*"[2]
- NO INFO: This treatment did not feature the information described above.

Based on the information above, this vignette experiment features a 2(within)×2(between) design, as illustrated in Figure S6. Besides randomizing allocation to the INFO and NO INFO-between conditions, we also randomized whether subjects first saw the EQUITY and then the INEQUITY condition or vice versa. In addition, we also randomize the order in which the punishment actions were displayed (from 0% to 100% punishment probability in either increasing or decreasing order). The experiment concluded with a demographic questionnaire and participants were then entered into a lottery with the chance to win $100 for participating.

Procedures

With the help of several research assistants, we collected data in person from a diverse population in May and June of 2021. Data was collected in various cities across 10 U.S. states, yielding a total of n=225 observations.[3] Our study was pre-registered (see Appendix C for details) and our analyses follow this pre-registration.

Results

Because our goal is to examine the robustness of punishment behavior observed in Study 1, both our hypotheses and analyses follow from it. To achieve comparability across settings, we

[1]We opted for testing only one norm nudge, and the NORMATIVE condition in particular, for the following two reasons: first, we wanted the norm information in the vignette to be based on truthful information. For practical reasons, we were able to verify this normatively but not empirically, given that it is a fictitious setting. The truthfulness of the normative message was obtained based on a sample (n=60) at the University of Pennsylvania. Participants were first shown the vignettes (using the exact wording) and then asked to indicate whether they believe that accepting a bribe is the right thing to do. A majority said that accepting a bribe is not the right thing to do. Second, as our behavioral results above indicate, the normative nudge leads directionally to lower punishment than the empirical nudge. For this reason, our normative nudge in the vignette may be understood as the lower-bound among the two nudges.

[2]The truthfulness of this message was obtained based on a student sample (n=60) at the University of Pennsylvania. Participants were first shown the vignettes (using the exact wording) and then asked to indicate whether they believe that accepting a bribe is the right thing to do. A majority of participants indicated that accepting a bribe is *not* the right thing to do. Details are available at https://osf.io/nfkyc.

[3]Various research assistants approached participants in the vicinity of city centers at random and explained that they could participate in a scientific experiment in return for which they would have the chance to win one of three $100 Amazon vouchers. Upon agreeing to participate, a QR code was presented that the participants were asked to scan on their phones. To ensure anonymity, the research assistants then stepped away and approached other potential participants. To be properly powered, we pre-registered to collect at least 200 observations.

Figure S7: Punishment probability chosen for accepting a bribe

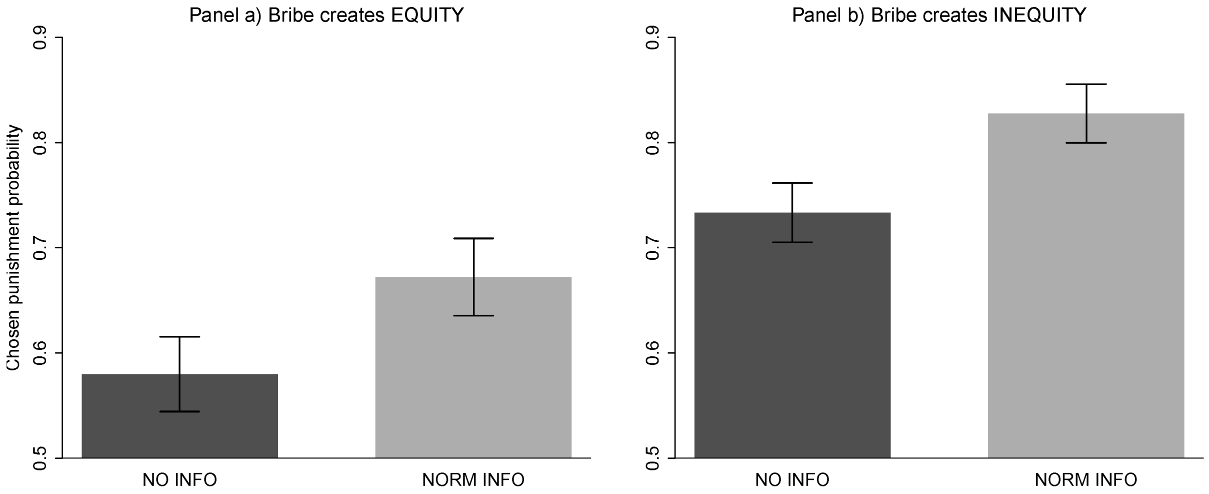

Notes: Punishment probability by whether NORM INFO or NO INFO is provided (between-subject design). Panels a) and b) show this for the situation when taking the bribe led to INEQUITY and EQUITY respectively (the two within-subject conditions). Error bars denote SEM.

are mainly interested in understanding two behavioral aspects: first, is enforcement sensitive to the provision of norm information? Second, do punishers enforce norms differently depending on whether Alex's acceptance of the bribe achieves equity versus inequity?

We first analyze the results using non-parametric tests. They mirror those from the behavioral experiment and are presented in Figure S7. For one, we observe that providing norm information significantly increases punishment for inequity-creating corruption (0.733 vs. 0.828, Wilcoxon rank-sum test: p=0.005) and, to a lesser extent, equity-creating corruption (0.672 vs. 0.580, p=0.074).[4] We also observe an increase when comparing the effect of norm information when examining the punishment formed as averages across the two different inequity scenarios (0.657 vs. 0.750, p=0.003). Consistent with the punishment behavior in Study 1, motives matter for the norm enforcement decisions across norm information treatments: the punishment for accepting an equity-creating bribe is always lower than that of an inequity-creating bribe (over both norm-INFO conditions 0.623 vs. 0.778, Wilcoxon signed-rank test: p<0.001). This also holds when we look at the (within-) effect of the bribe's equity-effect separately by whether norm information was provided to the subject (0.672 vs. 0.828, p<0.001) or not (0.580 vs. 0.733, p=0.002).

We also estimate the following regression model:

$$p_{is} = \alpha + \beta_1 \text{ INFO}_i + \beta_2 \text{ EQUITY-creating}_s + \boldsymbol{\gamma}' \, \textbf{Controls}_\textbf{i} + \epsilon_{is}. \tag{2}$$

[4]We also find a positive, significant effect of providing norm information when we only look at the first responses by subjects, before they experienced their respective within variation (0.645 vs. 0.762, p=0.010).

In the above, the dependent variable p_{is} is the chosen punishment probability of subject i in (equity-) scenario s (in %). INFO_i indicates whether the subject was in a treatment where norm information was provided and EQUITY-creating_s denotes whether accepting the bribe in scenario s created equity (as opposed to inequity). Finally, the vector $\mathbf{Controls_i}$ captures subjects' age and gender. It also contains a dummy to control for the order in which the two equity-scenarios were presented for each subject and whether the punishment options were presented in an increasing or decreasing order.

The results from estimating the above model by OLS (clustering standard errors on the subject level) are presented in Table S7. Column 1 shows the significant effects of providing norm-INFO and being in the EQUITY-creating scenario (8.1 ppt.-increase and 15.4 ppt.-decreases in punishment, respectively). Column 2 adds the interaction term of the two treatment dummies to the above model. The unchanged estimates for the non-interacted coefficients and the effectively zero coefficient for the interaction term show that the effect of providing the norm-INFO separately does not differ substantially across the equity-conditions. Overall, these findings corroborate our findings from the behavioral experiment in the context of whistle-blowing. Across different specifications, we find that the punishment for accepting a bribe is stronger when NORM-Info is provided and weaker when accepting the bribe creates equity.

Table S7: Punishment in the vignette experiment – regression results

	Punishment probability in %	
	(1)	(2)
INFO	8.120**	8.235**
	(4.027)	(3.927)
EQUITY	-15.444***	-15.336***
	(1.922)	(2.725)
INFO×EQUITY		-0.230
		(6.263)
Constant	64.159***	64.105***
	(7.458)	(7.439)
Controls	yes	yes
N	450	450
R^2	0.137	0.137

Notes: OLS results regressing the chosen punishment probability on a dummy whether a subject is in the norm-INFO treatment and whether the punishment refers to the situation with a EQUITY-creating bribe plus the interaction of the two; the baseline category is, therefore, a subject in treatment NO INFO and an inequity-creating bribe. Additional control variables include age, gender, and controls for how punishment scenarios were presented (increasing/decreasing) and whether the EQUTIY-creating was shown first. Two punishment scenarios per punisher; standard errors are clustered at the punisher level. */**/***: p<0.10/0.05/0.01.

Appendix C: Information regarding pre-registration

Both experiments were pre-registered on `http://aspredicted.org`. Below are the links to the anonymized pre-registration files that are uploaded to https://osf.io/nfkyc.

Links:

- Study 1 – Behavioral Experiment: https://osf.io/a3nzr
- Study 2 – Vignette Experiment: https://osf.io/4h7xk
- Study 3: https://osf.io/jnc65

Notes regarding data collection for the behavioral experiment of Study 1:

- Data for the behavioral experiment was collected in April, May, and November 2019.
- A first batch of 106 observations was collected in April 2016 to test the functionality of the experimental interface with subjects from the representative pool provided by Dynata. Based on these observations, we pre-registered the study and started to collect additional observations in May.
- Due to miscommunication with the data collection company, we obtained almost twice as many observations (987 instead of the targeted 500) for the punishers by May.
- Upon inspecting the data, we found that the quota-based sample was representative of the US population across our defined age and gender bins but not for the cross product of those bins (e.g., 50-59 years old females). We then approached the survey firm to rectify this issue. By a courtesy agreement, the firm sampled additional observations in November in order to have a representative sample also along these cross bins. This yielded our final sample with a total of 1,240 observations.
- Given this windfall in statistical power, the resulting improvement in the precision of our estimates (see, e.g., Maxwell et al., 2008), and to avoid a waste of resources, we decided to utilize the full sample.
- As a robustness check, we also repeated our analysis while excluding the 106 observations collected in April before we pre-registered the behavioral experiment. All results are similar in terms of significance and magnitude to those stated in the main text.
- For all punisher observations, we collected observations for the potentially punished active players according to our stated 15:1-ratio-rule (see Footnote 8).

Appendix D: Instructions

In the following, we display screenshots of all of our experiments. Specifically, we present them in the following order:

1. Instructions for the Liar (Sub-)Experiment in Study 1
2. Instructions for the Punisher (Sub-)Experiment in Study 1
3. Instructions for the Vignette Experiment in Study 2
4. Instructions for the experiment in Study 3

Notes:

- In order to facilitate a comprehensive display, we omit repeating elements such as "continue"-buttons.
- If a screen does not fit a single page in this appendix, it is split over two pages and the screen number gets ab an "a" or "b"-suffix for the first and second part of the screen (e.g., "Screen 7a).

All screenshots are uploaded to OSF at: https://osf.io/nfkyc
